# Identification and characterization of senescent macrophages in renal allograft rejection: a cross-species MultiOmics study

**DOI:** 10.3389/fimmu.2025.1623124

**Published:** 2025-10-09

**Authors:** Hanyu Xiao, Jie Zhang, Qidan Pang, Chengjun Yu, Jun Pei, Huyu Wang, Sheng Wen, Chunlan Long, Yi Hua, Guanghui Wei

**Affiliations:** ^1^ Department of Urology, Children’s Hospital of Chongqing Medical University, National Clinical Research Center for Child Health and Disorders, Ministry of Education Key Laboratory of Child Development and Disorders, Chongqing, China; ^2^ Chongqing Key Laboratory of Structural Birth Defect and Reconstruction, Chongqing, China; ^3^ Children Urogenital Development and Tissue Engineering of Chongqing Education Commission of China, Chongqing, China; ^4^ Department of General Surgery/Gastrointestinal Surgery, Bishan Hospital of Chongqing Medical University, Bishan Hospital of Chongqing, Chongqing, China; ^5^ Department of Nephrology, Bishan Hospital of Chongqing Medical University, Bishan Hospital of Chongqing, Chongqing, China

**Keywords:** cellular senescence, macrophages, kidney transplantation, allograft rejection, T-cell mediate rejection

## Abstract

**Background:**

Allograft rejection remains a main hindrance for long-term graft survival. Cellular senescence (CS) contributes to graft injury, but the role of immune cell senescence in rejection remains unclear.

**Methods:**

Microarray data from renal transplant biopsy cohorts and age-matched rat allograft models were integrated to characterize senescence phenotypes. Immune cell infiltration algorithms and histopathology were employed to recognize major senescent alloimmune subpopulation. Then, novel senescent infiltrating macrophages (SnIMs) were identified using cross-species single-cell transcriptomics and validated in rat models. Finally, the clinical values of SnIMs were evaluated in renal transplant datasets.

**Results:**

CS gene sets were enriched in rejecting allografts, correlating with graft loss and pathological injury. Alloimmune responses amplified stress-induced senescence in rat allografts, with p21+ macrophages emerging as the important senescent immune subtype. SnIMs exhibited cell cycle arrest, upregulation of senescence-associated secretory phenotype, and conserved transcriptional signatures driven by NF-κB/Cebpb across species through single-cell analysis. These cells accumulated along pseudotime during rejection and interacted with effector T cells via CXCL chemokines. Clinically, SnIM infiltration predicted T cell–mediated rejection and correlated with Banff lesion grades and poor graft survival.

**Conclusions:**

Our findings identify novel stress-induced SnIMs in renal allograft rejection and highlight their pathogenic role in rejection injury, providing a therapeutic target to improve renal transplant outcome.

## 1 Introduction

Despite remarkable progress in immunosuppressive therapies, allograft rejection remains a main hindrance for long-term graft survival (1). In renal transplantation, the incidence of rejection persists at approximately 20% within the first postoperative year, adversely impacting patient outcomes and imposing a burden on public health (2). Current pathological classification categorizes rejection into two primary types: T cell-mediated rejection (TCMR) and antibody-mediated rejection (AMR). The mechanisms of both types are complex involving aberrant activation of adaptive immune responses and synergistic engagement of innate immune components (3). Unfortunately, existing immunosuppressants that target these established pathways fail to produce satisfactory results in clinical practice. Thus, we need to explore novel regulatory mechanisms of alloimmune injury in hope of uncovering potential therapeutic targets.

Cellular senescence (CS) is an irreversible process triggered by external and internal stress, resulting in permanent cell cycle arrest (4). In general, CS is divided into two types: replicative senescence and premature senescence. Replicative senescence arises secondary to telomere shortening and is primarily observed in physiological contexts such as natural aging. In contrast, premature senescence refers to senescence induced by diverse stress stimuli, including oxidative stress, DNA damage, mitochondrial dysfunction, inflammation, and oncogene activation, collectively termed stress-induced premature senescence (SIPS) (5).

Recent studies suggest that senescent cell accumulation in elderly donor kidneys strongly correlates with post-transplant interstitial fibrosis, tubular atrophy, and chronic allograft nephropathy (CAN) progression (6). Elevated pre-transplant mRNA levels of senescence markers (p21^Cip1^ and p16^Ink4a^) in biopsies are an independent risk factor of poor graft outcomes (7). Mechanistically, senescent cells mediate inflammatory cascades within the graft microenvironment through senescence-associated secretory phenotype (SASP)—a complex mixture of pro-inflammatory cytokines, chemokines, and matrix-remodeling proteases. This SASP-driven process ultimately compromises renal regenerative capacity and accelerates fibrotic remodeling (8). Targeted clearance of donor senescent cells pre-transplantation shows therapeutic potential for improving graft survival (9).

Although the detrimental impact of donor age-related senescence on transplant outcomes is well-established, the pathophysiological interplay between SIPS and alloimmunity, particularly allograft rejection, remains incompletely elucidated. Notably, the dynamic of alloimmune microenvironment (AME) may exacerbate senescence phenotypes through dual mechanisms: On one hand, immunosuppression compromises the host’s capacity to clear senescent cells (10); on the other hand, transplantation-associated stressors, including inevitable ischemia-reperfusion injury (IRI) and persistent alloimmune response, aggravate senescent cell accumulation, as evidenced by aberrantly high p16^Ink4a^ and p27^Kip1^ expression in rejection biopsies (11). Emerging evidence suggests that senescent immune cells drive senescence and tissue injury in solid organs (12). We thus posit that immune cell senescence constitutes a potential driver in allograft rejection injury.

Conventional senescence biomarkers, limited by insufficient specificity, fail to distinguish activated immune cells from senescent cells. Transcriptomics, with its high-throughput capacity, overcomes the limitations of single-marker approaches for senescence characterization (13). Single-cell transcriptome enables precise evaluation of CS state at cellular resolution by detecting senescence-specific transcriptional profiles. This study integrates multi-omics data with renal transplant experimental models to explore the role of CS in rejection pathogenesis. Crucially, we manage to identify stress-induced senescent infiltrating macrophages (SnIMs) as the major immune senescence subtype in rejection and their cross-species genetic markers through single-cell analysis. Moreover, we observed that the burden of SnIMs infiltration correlates with poor graft outcomes and Banff lesion severity of TCMR in kidney transplant biopsy cohorts. In summary, our findings provide evidence for post-transplant senolytic therapies.

## 2 Materials and methods

### 2.1 Data acquisition

This study integrated multiple kidney transplant transcriptomic datasets from the Gene Expression Omnibus database (https://www.ncbi.nlm.nih.gov/geo/). Microarray data were derived from two consecutive renal transplant cohorts: the prognostic cohort (GSE21374 (14), n=282 with graft survival follow-up) and the Banff cohort (GSE98320 (53, 15), n=685 with histopathological injury scores). All participants underwent indicated biopsies with diagnostic confirmation by independent pathologists using the Banff criteria (**Supplementary Table S1**). Additionally, in the Banff cohort, we excluded samples with incomplete Banff score information.

Single-cell transcriptomic data encompassed cross-species analyses: Murine datasets: Dataset 1 [GSE252337 (15)] included BALB/c→C57BL/6 allografts (rejection group n=2, naïve controls n=2); Dataset 2 [GSE157292 (15, 16)] utilized DBA/2J→C57BL/6 transplant (rejection n=1). Human dataset [GSE189536 (17)] integrated 11 indicated biopsies, covering AMR (n=4), TCMR (n=3), mixed rejection (n=2), and non-rejection controls (n=2).

*In vitro* activated macrophage RNA-seq data were derived from GSE267544 (18), which contains gene expression profiles of mouse bone marrow-derived macrophages (BMDMs) activated via the classical LPS/IFN-γ pathway.

CS gene sets/signatures were curated from the Molecular Signatures Database (MSigDB) (https://www.gsea-msigdb.org/gsea/index.jsp) and supplemented with high-impact literature-derived signatures (**Supplementary Tables S2**, **Table S3**).

### 2.2 Data processing and analysis

Microarray data Analysis: Raw data were log2-transformed and batch-corrected during processing. Probe IDs were mapped to gene symbols. Enrichment of CS gene sets across groups was evaluated using Gene Set Enrichment Analysis (GSEA) (19). Gene set variation analysis (GSVA) (20) was conducted to compute CS scores. Samples were stratified into “High score” and “Low score” groups based on the median value of each CS scores across the entire cohort, and then survival differences were assessed using Kaplan-Meier curves with log-rank tests between groups. For immune infiltration, single-sample GSEA (ssGSEA) was used to quantify 22 immune cell signatures (21) in AME, followed by Pearson correlation analysis with CS GSVA scores.

Single cell RNA-seq Analysis: Data were processed using Seurat (v4.2). Quality control was performed to retain cells expressing 200-5,000 genes with mitochondrial content <25%. Log-normalization and variance-stabilizing transformation were applied to identify the top 2,000 variable genes. Harmony (22) integration was used for batch effect correction, followed by PCA, Louvain clustering (resolution = 0.5), and UMAP visualization. Cluster-specific markers were identified via Wilcoxon rank-sum tests (*p* < 0.05, *log2FC* > 0.25) and annotated using CellMarker2.0 (23).

Senescent Cell Identification Tool (SnCIT): While the SenMayo signature (24), recommended by the SenNet Consortium, was used for senescence detection at single-cell resolution (13), its exclusion of canonical markers (*CDKN1A/CDKN2A*) necessitated supplementation with the Martina-defined Senescence signature (*Cdkn2a, Cdkn1a, Serpine1, Cdkn1b, Cdkn2d, Cdkn2b*) *(* 25) to mitigate SASP-centric bias and reduce false positives. Feature expression of these two selected signatures was scored using AddModuleScore (Seurat package) in each cell. Cluster cells scoring in the top 25% for both signatures were classified as senescent. Senescence features were confirmed via ssGSEA, cell cycle analysis (CellCycleScoring), and differential gene expression (Wilcoxon test; *p* < 0.01, *logFC* > 0.5). Enriched pathways and transcription factors were analyzed using Metascape (26).

Trajectory and Cell-Cell Communication: Differentiation trajectories were reconstructed using Monocle2 (27) via high-variance genes and DDRTree reduction. Differentiation potential was inferred using CytoTRACE2 (28). Ligand-receptor signaling networks were analyzed using CellChat (29).

### 2.3 Animal models

A rat renal transplantation model was established using 8- to 10-week-old male Sprague-Dawley (SD; n=12, Chongqing Medical University, SYXK[YU]2022-0016) and Wistar rats (n=4; Beijing Vital River Laboratory Animal Technology Co., Ltd., SCXK[Jing]2021-0006). Allogeneic grafts (Allo group: SD-to-Wistar) and syngeneic controls (Syn group: SD-to-SD) were orthotopically transplanted under intraperitoneal anesthesia with 2% sodium pentobarbital (40 mg/kg). Specifically, donor left kidneys were perfused with heparinized saline (1% w/v), excised, and cold-preserved at 4°C, and transplanted into recipients after native left nephrectomy. Vascular anastomoses (renal artery and vein) and ureteroneocystostomy were performed in the retroperitoneal cavity using 10–0 nylon sutures in a continuous pattern, with the recipient’s right kidney retained. No immunosuppressants were administered postoperatively. All recipient rats survived until postoperative day 7, when euthanasia was performed via intraperitoneal injection of sodium pentobarbital (150 mg/kg), with kidney tissues either fixed in 4% paraformaldehyde for histology or snap-frozen in liquid nitrogen (−80°C) for protein analysis. Procedures adhered to protocols approved by the Children’s Hospital of Chongqing Medical University Animal Ethics Committee (CHCMU-IACUC20220429006).

### 2.4 Hematoxylin and eosin staining

Renal specimens were fixed in 10% neutral buffered formalin (24 h), dehydrated through graded ethanol, and paraffin-embedded. Sections were cut, baked at 60°C, deparaffinized, and rehydrated. Nuclear staining used Harris’s hematoxylin (5 min), differentiated with 1% acid alcohol, and blued in tap water. Cytoplasmic counterstaining employed eosin Y (1 min). After ethanol dehydration and xylene clearing, sections were mounted with neutral resin. Stained slides were analyzed under a bright-field microscope.

### 2.5 Senescence-associated β-galactosidase staining

Following the manufacturer’s protocol (Senescence β-Galactosidase Staining Kit, Servicebio G1073-100T), fresh frozen kidney sections were air-dried, fixed with β-galactosidase fixative, and PBS-washed. A working solution (940 μL Solution A, 10 μL Solution B, 50 μL X-Gal) was applied, and sections were incubated at 37°C for 16–18 h. post-incubation, slides were rinsed in PBS and distilled water, counterstained with Nuclear Fast Red, dehydrated, and mounted. Blue cytoplasmic precipitates (β-galactosidase activity) and red nuclei were imaged via bright-field microscopy.

### 2.6 Immunofluorescence staining

Paraffin-embedded kidney sections were dewaxed, rehydrated through ethanol gradients, and subjected to microwave-based antigen retrieval in EDTA buffer. After endogenous peroxidase blockade and serum blocking, primary antibodies were incubated overnight at 4°C. HRP-conjugated secondary antibodies and tyramide signal amplification (TSA) were sequentially applied. Multiplex detection was achieved through iterative cycles of antibody stripping, re-blocking, and reprobing. Nuclei were counterstained with DAPI. Images were acquired using confocal microscopy.

### 2.7 Immunohistochemistry

Deparaffinized sections were rehydrated through graded alcohols to water. Antigen retrieval utilized heat-mediated unmasking. Endogenous peroxidase activity was quenched with 3% H_2_O_2_ (10 min). Sections were circled with a hydrophobic barrier and blocked with normal serum (30 min). Primary antibody incubation proceeded overnight at 4°C. After washes, sections received HRP-conjugated secondary antibody (RT, 30 min). Antibody binding was visualized using DAB chromogen. Nuclei were counterstained with hematoxylin. Sections underwent dehydration, clearing in xylene, and mounting prior to microscopy.

### 2.8 Transplant transcriptomics

Allo/Syn grafts (each n = 4) RNA was sequenced on Illumina NovaseqTM 6000 (LC-Bio Technology CO., Ltd., Hangzhou, China). Trimmomatic-filtered reads were aligned (HISAT2, GRCh38) and quantified (StringTie). FPKM normalization incorporated gene length and total mapped fragments.

### 2.9 Statistical analysis

Analyses were performed in R (v4.2) and GraphPad Prism 10. Quantitative features of imaging data were analyzed using ImageJ. All metric data underwent initial verification through Shapiro-Wilk normality testing and Levene’s variance homogeneity assessment. Subsequent comparative analyses between study groups were conducted using either Student’s t-test (parametric conditions) or the Mann-Whitney U test (non-parametric conditions), with statistical significance threshold set at *α* = 0.05.

## 3 Results

### 3.1 Cellular senescence is a characteristic phenotype in renal allograft rejection

**Figure 1:**
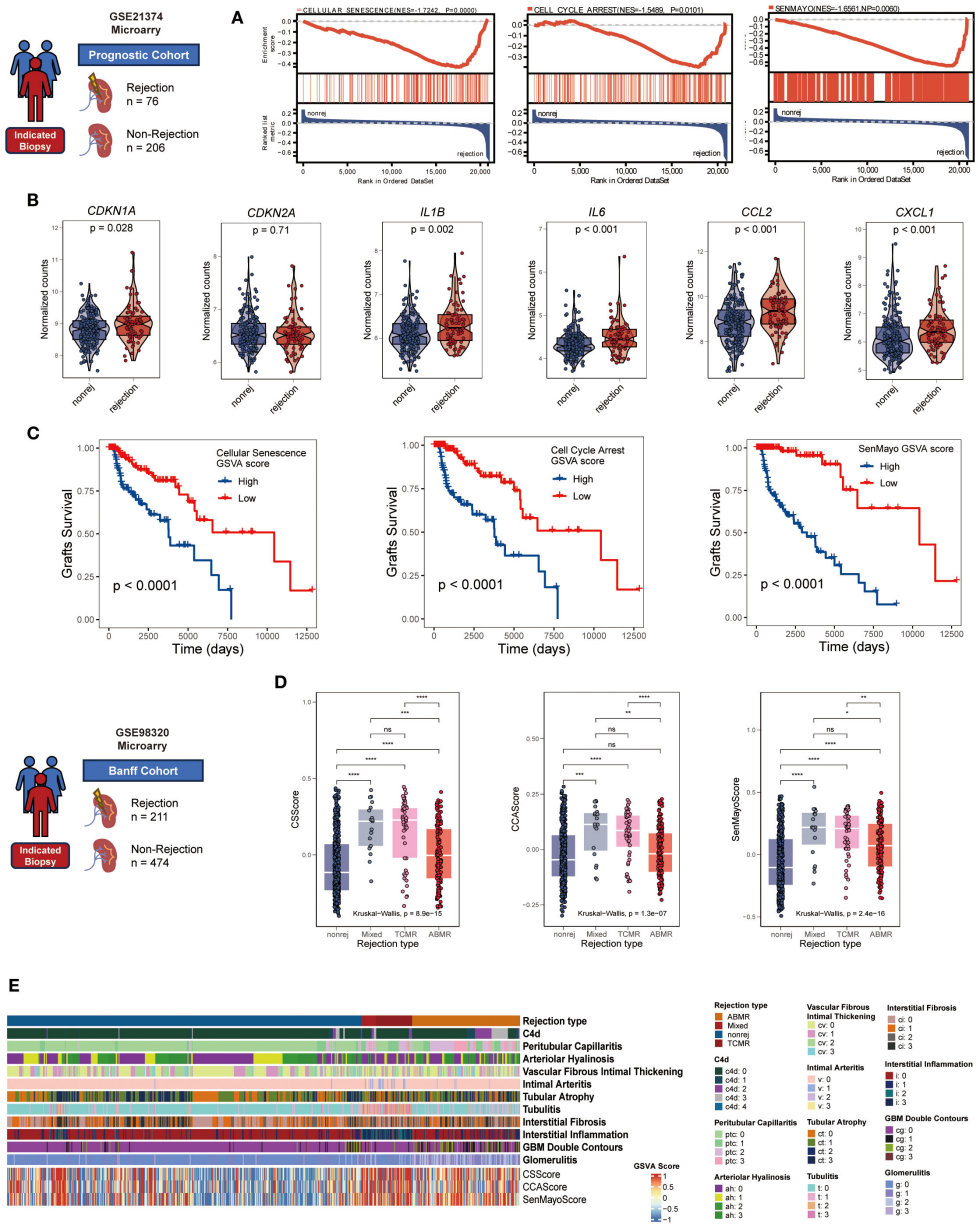
Cellular senescence characterizes renal allograft rejection in clinical cohorts. **(A)** GSEA plots manifesting significant enrichment of senescence-associated gene sets (GOBP: CELLULAR SENESCENCE, GOBP: CELL CYCLE ARREST, and SenMayo) in rejection versus non-rejection biopsies (all *NP* < 0.05, normalized enrichment score >1.5). **(B)** Violin plots comparing mRNA expression of core senescence markers (*CDKN1A, CDKN2A*) and SASP components (*IL1B, IL6, CCL2, CXCL1*) between rejection and non-rejection groups (all *p* < 0.05 except *CDKN2A*, unpaired two-tailed t-test). **(C)** Kaplan-Meier curves showing inferior graft survival in patients with high cellular senescence GSVA scores (log-rank all *p* < 0.001). **(D)** Comparative cellular senescence GSVA scores across rejection subtypes: antibody-mediated rejection (AMR), T cell-mediated rejection (TCMR), mixed rejection, and non-rejection controls (Kruskal-Wallis test, all *p* < 0.001). **(E)** Heatmap illustrating coordinated elevation of senescence pathway activities with progressive Banff lesion severity (rows: senescence signatures, Banff lesion classification, rejection types; columns: samples; color scale: z-scored GSVA scores). *p<0.05, **p<0.01,***p<0.001,****p<0.0001.

To explore the role of CS in renal allograft rejection, we integrated transcriptomic datasets from for-cause renal biopsy samples of two independent renal transplant cohorts. In the prognostic cohort, significant enrichment of three CS gene sets/signatures (REACTOME: CELLULAR SENESCENCE, GOBP: CELL CYCLE ARREST, and SenMayo) was observed in rejection samples using GSEA analysis (**Figure 1A**). Notably, 60-90% of the genes across the various CS gene sets were not included in the B-HOT panel (30) (**Supplementary Table S3**)—an established gene panel of rejection—supporting the notion that​ these gene sets provide more specific insights into senescence-related mechanisms beyond conventional rejection pathways. Furthermore, mRNA expression levels of canonical CS markers (*CDKN1A* rather than *CDKN2A*) and core SASP components (*IL1B, IL6, CCL2, CXCL1*) were consistently upregulated in rejecting allografts (**Figure 1B**). These upregulations were validated ​across the multiple independent cohorts​ integrated within the PROMAD atlas (**Supplementary Figure S1**) (31). Through survival analysis, grafts with higher CS GSVA scores exhibited elevated risks of graft loss (**Figure 1C**). When testing the enrichment of CS gene sets in the Banff cohort, the GSVA scores were significantly higher in TCMR and mixed rejection samples and correlated positively with Banff lesion scores (**Figures 1D**, **E**). Notably, compared to AMR, the GSVA scores exhibited significantly greater differences among Banff lesion grades associated with TCMR: interstitial inflammation (i), intimal arteritis (v), tubulitis (t), interstitial fibrosis (ci), tubular atrophy (ct), demonstrating a graded positive correlation (**Supplementary Figure S2**). Given all that, we have good reasons to believe that CS is a characteristic phenotype in renal allograft rejection, especially TCMR.

### 3.2 Stress-induced senescence is aggravated in renal allograft rejection

**Figure 2:**
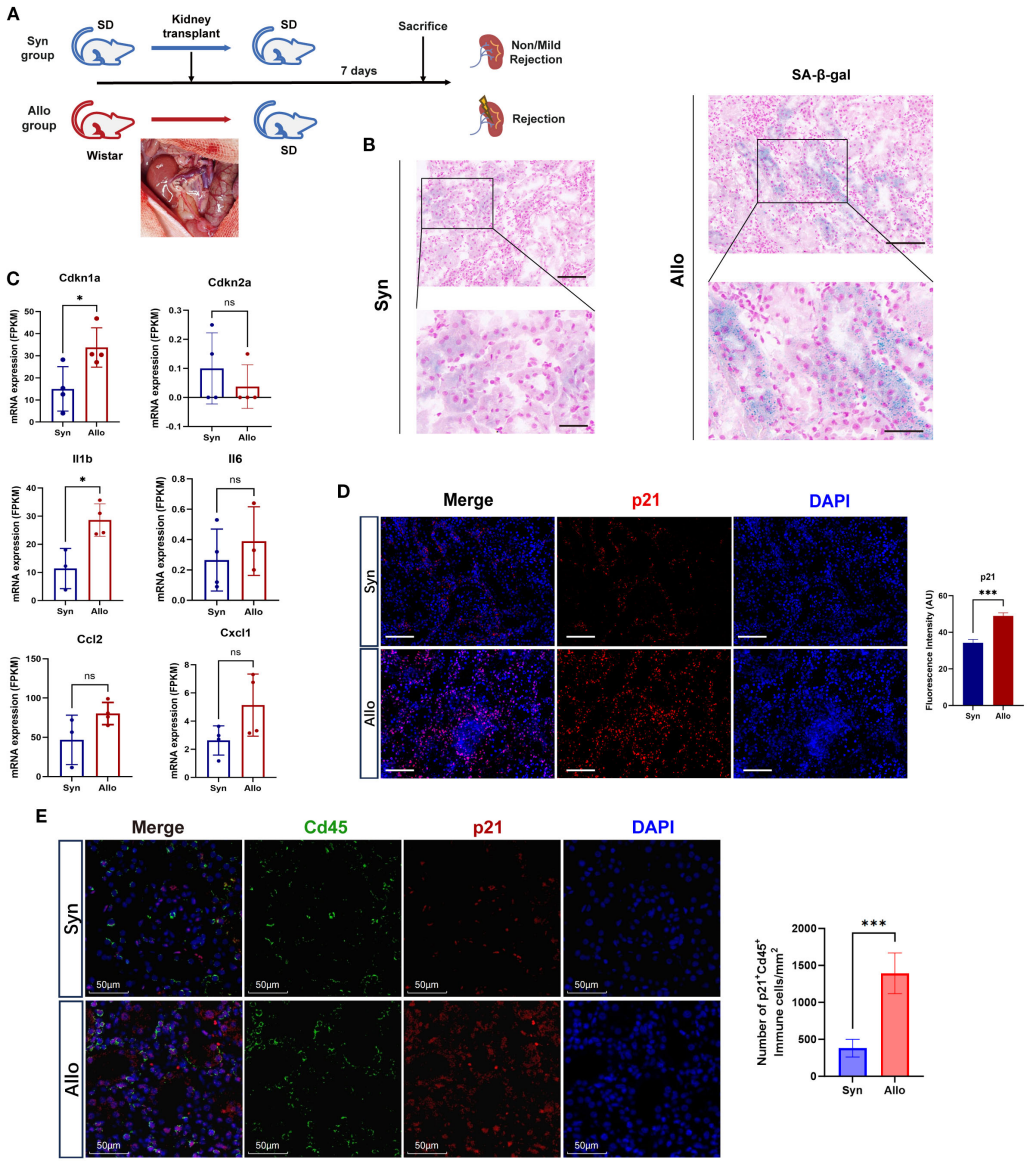
Cellular senescence biomarkers are elevated in allograft rejection of the rat model. **(A)** Schematic workflow of syngeneic (Syn) versus allogeneic (Allo) rat orthotopic kidney transplantation (n=4/group). Inset: Surgical image of graft implantation. **(B)** Representative images of SA-β-Gal staining (blue) in the Syn, and Allo groups on postoperative day 7 (Scale bar 100 μm, partial enlarged drawing with Scale bar 20 μm). **(C)** Elevated mRNA levels (FPKM) of senescence markers (*Cdkn1a, Cdkn2a*) and SASP components (*Il1b, Il6, Ccl2, Cxcl1*) in Allo versus Syn groups (*p < 0.05, unpaired two-tailed t-test). **(D)** Immunofluorescence co-staining of p21 (red, expressed in both the nucleus and cytoplasm) and nuclei (DAPI, blue) in the Syn and Allo groups (Scale bar 100μm). Barplot showing the fluorescence intensity of p21 in Allo versus Syn groups (***p < 0.001, unpaired two-tailed t-test); AU, Arbitrary Units. **(E)** Immunofluorescenceco-staining for p21 (red) with Immune cell marker Cd45 (green) in Syn and Allo groups (Scale bar 50μm). Barplot showing the number of p21^+^Cd45^+^ immune cells in Allo versus Syn groups per 1mm^2^ (***p < 0.001, unpaired two-tailed t-test).

Age-matched rat renal transplant models were established to avoid confounding factors such as donor-recipient age and storage time of grafts existing in the cohorts (**Figure 2A**). H&E staining showed that Allo group displayed distinctly exacerbated acute rejection injury, characterized by extensive interstitial infiltration of immune cells, though both groups exhibited IRI features (tubular injury) (**Supplementary Figure S4A**). Importantly, more remarkable senescence phenotype was observed in Allo group, manifested by intensified SA-β-gal staining (**Figure 2B**) and upregulated expression of *Cdkn1a* (encoding p21) as well as SASP genes (**Figure 2C**). Notably, the expression of *Cdkn2a* (encoding p16) didn’t show similar tendency, which was likely attributed to transcript dropout during sequencing. p21 was overexpressed in Allo group compared with Syn group, confirmed by Immunofluorescence and validated by IHC (**Figure 2D**, **Supplementary Figure S4A**). Regionally, the peritubular area exhibited a substantially greater proportion of p21-positive IHC staining than glomeruli (**Supplementary Figure S4A**). These findings suggest more pronounced senescence features in tubular and tubulointerstitial compartments during rejection, an observation further validated through β-galactosidase staining (**Supplementary Figure S4B**) and immunofluorescence assays (**Supplementary Figure S4C**). Furthermore, to delineate cell-type-specific senescence, we performed double immunofluorescence co-staining for p21 with tubular marker Lrp2 and immune marker Cd45. Quantitative analysis revealed no significant difference in p21^+^Lrp2^+^ tubular cell counts between Allo and Syn groups (P > 0.05) (**Supplementary Figure S5**). Conversely, p21^+^Cd45^+^ immune cells were significantly elevated in the Allo group (P < 0.001) (**Figure 2E**). These results collectively indicated that stress-induced senescence was activated at the early stage of acute rejection mediated by alloimmune responses.

### 3.3 Macrophage senescence is an important component of immune senescence in rejection

**Figure 3:**
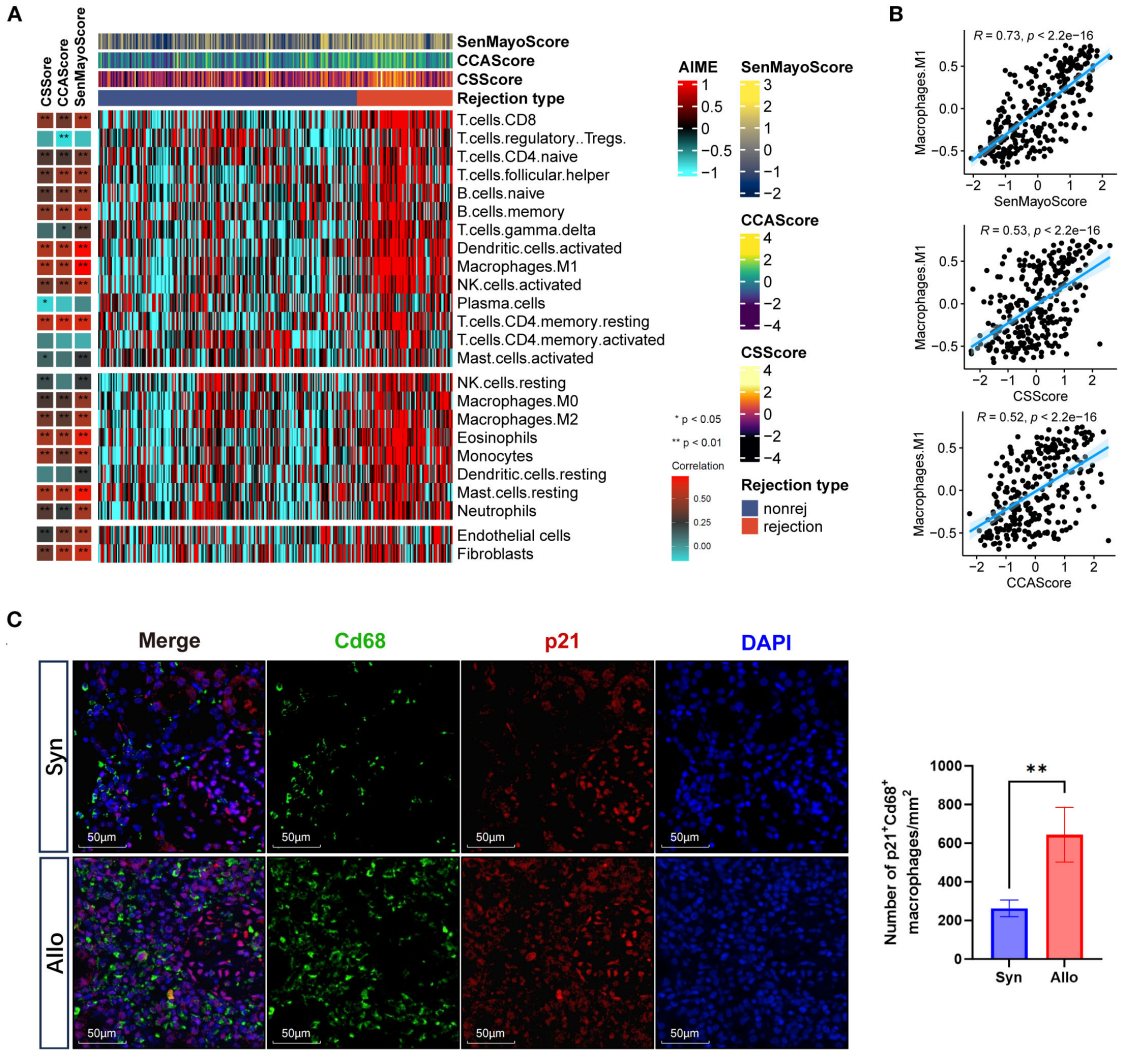
Cellular senescence is associated with macrophages in graft rejection. **(A)** Heatmaps showing the immune profile in the prognostic cohort, with the right panel showing the enrichment level of 24 cell types in alloimmune microenvironment and the left panel showing their correlation with three senescence GSVA scores (CS, CELLULAR SENESCENCE; CCA, CELL CYCLE ARREST; SenMayo). The senescence GSVA scores and rejection types were annotated at the top of the heatmap. **(B)** Scatter plots showing the correlation between inflammatory macrophages (M1) infiltration and three senescence GSVA scores (Pearson’s test). The blue regression curve represents the fitted linear relationship between variables, and the blue shaded areas on both sides of the curve indicate the 95% confidence interval. **(C)** Immunofluorescence co-staining of p21 (red, expressed in both the nucleus and cytoplasm) and Cd68 (green, expressed on the cell membrane) and nuclei (DAPI, blue) in the Syn and Allo groups (Scale bar 100 μm). *p<0.05, **p<0.01,***p<0.001,****p<0.0001.

The overexpression of p21 within infiltrating immune cells in allografts prompted the hypothesis that rejection induces senescence in immune cells. To verify this hypothesis, we conducted immune infiltration analysis in the prognostic cohort using ssGSEA. Analysis revealed that the CS GSVA score showed significant positive correlations with infiltration levels of effector immune cells in rejection responses, including CD4+ T cells, CD8+ T cells, NK cells, macrophages, B cells, dendritic cells, neutrophils, and mast cells (**Figure 3A**, **Supplementary Figure S6**). Notably, the highest correlation coefficient was observed between SenMayoScore and M1 macrophages (R = 0.73, *p* < 2.2 × 10^−16) (**Figure 3B**). This association was experimentally validated by immunofluorescence manifesting enhanced co-localization of macrophage marker CD68 (green) with p21 signals (red) in the Allo group (**Figure 3C**). Moreover, compared to M0 and M2 macrophage subtypes, M1 macrophage infiltration demonstrated significantly stronger correlations with the CS GSVA scores (**Supplementary Figure S7**). These findings implicate proinflammatory macrophages (M1) as the significant senescent immune cell population in rejection.

### 3.4 Identification of senescent macrophages in renal allograft rejection

To identify senescent macrophages in rejection, we analyzed single-cell datasets from two murine and one human renal allograft rejection study. In mouse dataset 1, all cells were partitioned into 20 subpopulations through dimensionality reduction and clustering (**Supplementary Figures S8A–C**), in which immune cell clusters distinctly enriched in rejection samples (**Supplementary Figure S8D**). The AddModuleScore algorithm revealed upregulated expressions of SenMayo and Senescence signature genes in rejection groups (**Supplementary Figure S8E**), and these genes were spatially enriched in macrophage subclusters (**Supplementary Figure S8F**), further supporting previous findings.

**Figure 4:**
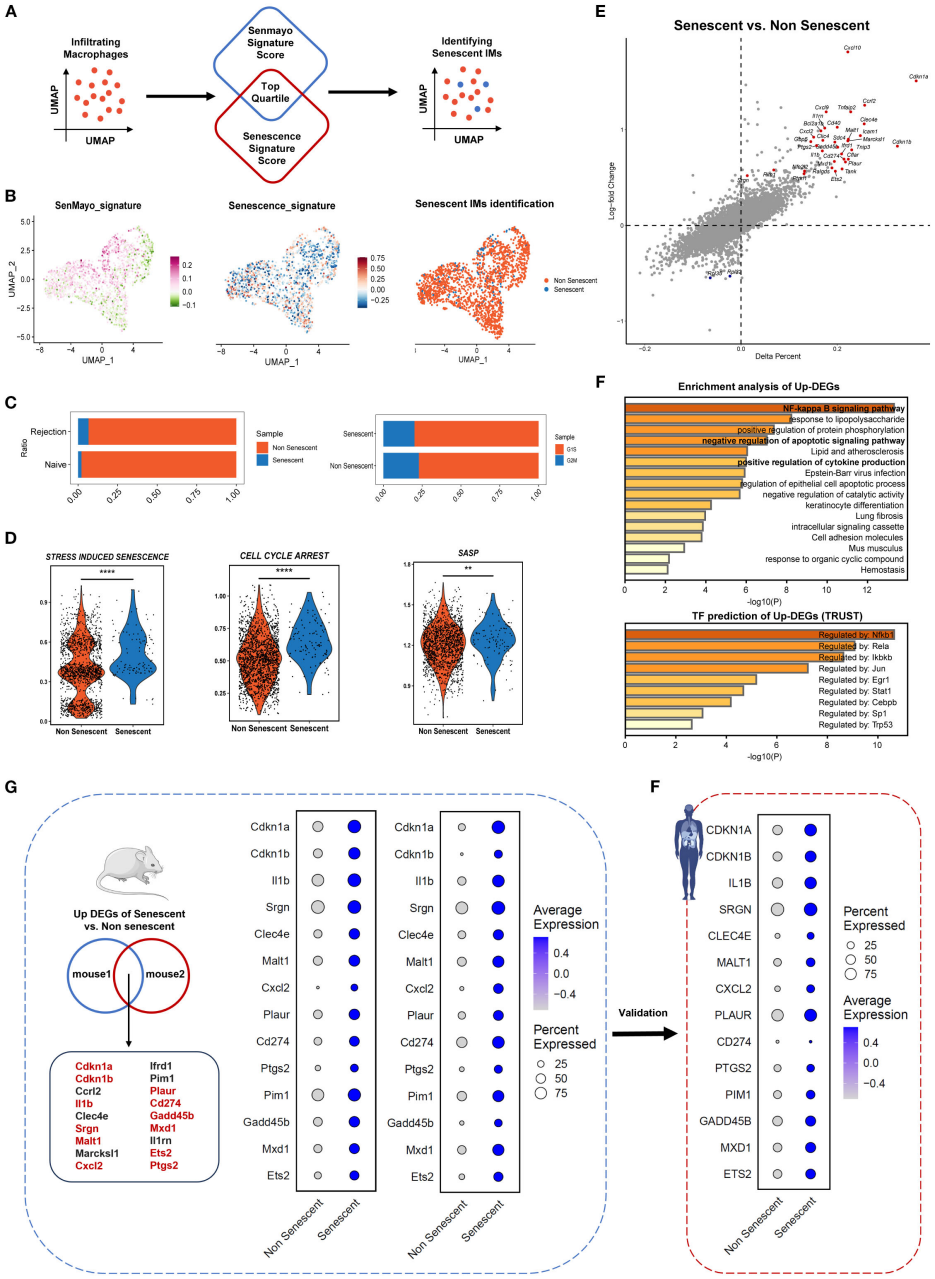
Identification of senescent infiltrating macrophages across species in kidney transplant rejection. **(A)** Schematic representation of Senescent Cell Identification Tool (SnCIT) for identifying senescent infiltrating macrophages (SnIMs) in macrophage cluster. **(B)** UMAP plots of infiltrating macrophages colored by SenMayo_signature, Senescence_signature and senescent cells. **(C)** Bar plots showing proportion of senescent and not senescent IMs between rejection and naïve samples, and G1/ S and G2/M cell cycle phases. D Violin plots showing ssGSVA scores of senescence gensets (GOBP: STRESS-INDUCED PREMATURE SENESCENCE, GOBP: CELL CYCLE ARREST, REACTOME: SENESCENCE-ASSOCIATED SECRETORYPHENOTYPE) of senescent and not senescent IMs (two sided Wilcox-test, **p < 0.01, **** *p* < 0.0001). **(E)** Dot plot indicating differentially expressed genes (DEGs) between senescent and not senescent IMs. Genes with a *p* < 0.01 and *logFC* > 0.5 are shown highlighted as upregulated (red) in senescent IMs. **(F)** Bar plots displaying enrichment analysis and transcription factor prediction of upregulated DEGs (up-DEGs) in senescent IMs, using Metascape and Trust database. **(G)** Venn diagram showing overlap of up-DEGs in SnIMs between the 2-mouse single-cell datasets. The genes highlighted in red have evidence to associate them with senescence. Dotplots demonstrating expression and percentage of intersecting up-DEGs in senescent and not senescent IMs of mouse datasets, which were validated in human dataset **(H)** and consistent across species.

Subsequent analyses focused on monocyte-derived infiltrating macrophages (IMs), identified as Cd68+ Plac8+ immune cells in renal tissues. First, IMs represent the dominant macrophage population driving inflammation during allograft rejection; Second, the senescence phenotypes of tissue-resident macrophages were partly attributed to homeostatic turnover (13). By integrating CS signature scoring, we successfully developed a computational framework (SnCIT) (**Figure 4A**) (**Supplementary 3**) that robustly identified *Cdkn1a*+ SnIMs (**Figure 4B**). SnIMs showed distinct enrichment in rejection samples, characterized by G1/ S-phase cell cycle arrest (**Figure 4C**), elevated activity of senescence pathways (GOBP: SIPS, GOBP: CELL CYCLE ARREST, REACTOME: SASP) (**Figure 4D**), and marked overexpression of: (i) Cell cycle inhibitors: *Cdkn1a, Cdkn1b, Gadd45b*; (ii) SASP components: *Cxcl10, Il1b, Cxcl2*; (iii) Anti-apoptotic mediators: *Bcl2a1b*; (iv) Senescence surface markers: *Icam1* (immune interaction), *Cd274*/PD-L1 (immune evasion), and *Plaur*/uPAR [senescence-specific membrane protein (32)] (**Figure 4E**). Furthermore, SnIM-upregulated differentially expressed genes (DEGs) enriched in NF-κB signaling, apoptosis resistance, and cytokine production pathways. NF-κB family members (Nfkb1, Rela, Ikbkb), Jun, Stat1, and Cebpb were implicated as key transcription factors (TFs) of SnIMs gene networks using TRUST database (**Figure 4F**). These pathways and TFs are established regulators of inflammatory SASP secretion and persistent senescence (13).

Additionally, we confirmed *Cdkn1a+/CDKN1A+* SnIMs with similar transcriptional features, molecular pathways and regulatory networks in mouse dataset 2 and human dataset (**Supplementary Figure S9**). A conserved transcriptional signature was yielded through intersection analysis of SnIM-upregulated DEGs from two murine datasets (**Figure 3G**, **Supplementary Table S4**), subsequently validated in human dataset (**Figure 3F**). To evaluate the specificity of SnIM signature, we analyzed its component genes in a publicly available dataset of *in vitro* stimulated BMDM. While the majority of signature genes (9/14) were significantly upregulated at 12 hours post M1-polarization (LPS/IFN-γ), the expression of most genes (e.g., *Malt1*, *ptgs2*) had returned to low levels by 24 hours, and core senescence markers *Cdkn1a* and *Cdkn1b* showed no significant induction at two time point (**Supplementary Figure S10**). This expression pattern demonstrates that the sustained transcriptional profile of SnIMs observed in allografts is not entirely consistent with a transient macrophage activation response and further supports its specificity to a senescence-like state.

**Figure 5:**
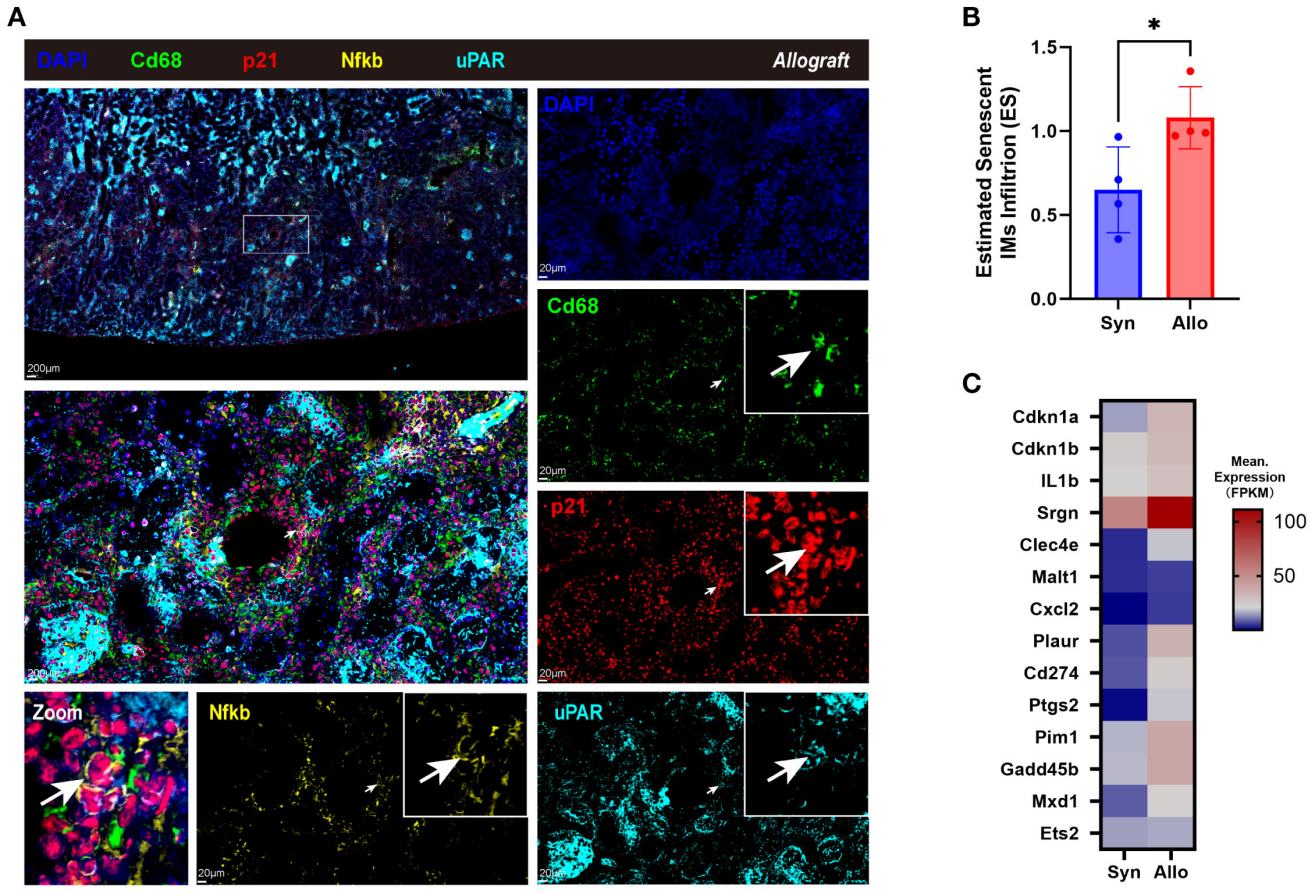
Identifying senescent infiltrating macrophages in allograft rejection of the rat model. **(A)** Immunofluorescence co-staining of Cd68 (green, expressed on the cell membrane), p21 (red, expressed in both the nucleus and cytoplasm), uPAR (cyan, expressed on the cell membrane), Nfkb (ginger, expressed in both the nucleus and cytoplasm), and nuclei (DAPI, blue) in cortex of allograft of the rat model (Scale bar 200 μm or 20 μm), with the white arrows pointing the senescent-like macrophages. **(B)** Estimated SnIMs infiltration using GSEA in Allo versus Syn grafts (*p < 0.05, unpaired two-tailed t-test; ES, Enrichment Score). **(C)** Heatmap showing conserved signature of SnIMs in Allo versus Syn grafts.

In rat allografts, senescent-like macrophages (Cd68+ p21+ uPAR+ Nfkb+) were localized to perivascular and tubulointerstitial regions by multiplex immunofluorescence (**Figure 5A**), presenting enlarged size and irregular shape (white arrows). SnIM infiltration (using *Plac8, Cd68* and SnIM signature as cell markers) was significantly higher in Allo than Syn groups (*p* < 0.05), estimated by ssGSEA (**Figure 5B**), which is consistent with the upregulation of SnIM signature genes (**Figure 5C**). These analyses suggested that there was a conserved SnIM subcluster across species in renal allograft rejection.

### 3.5 Senescent macrophages accumulated as rejection progressed and showed interacted with T cells

**Figure 6:**
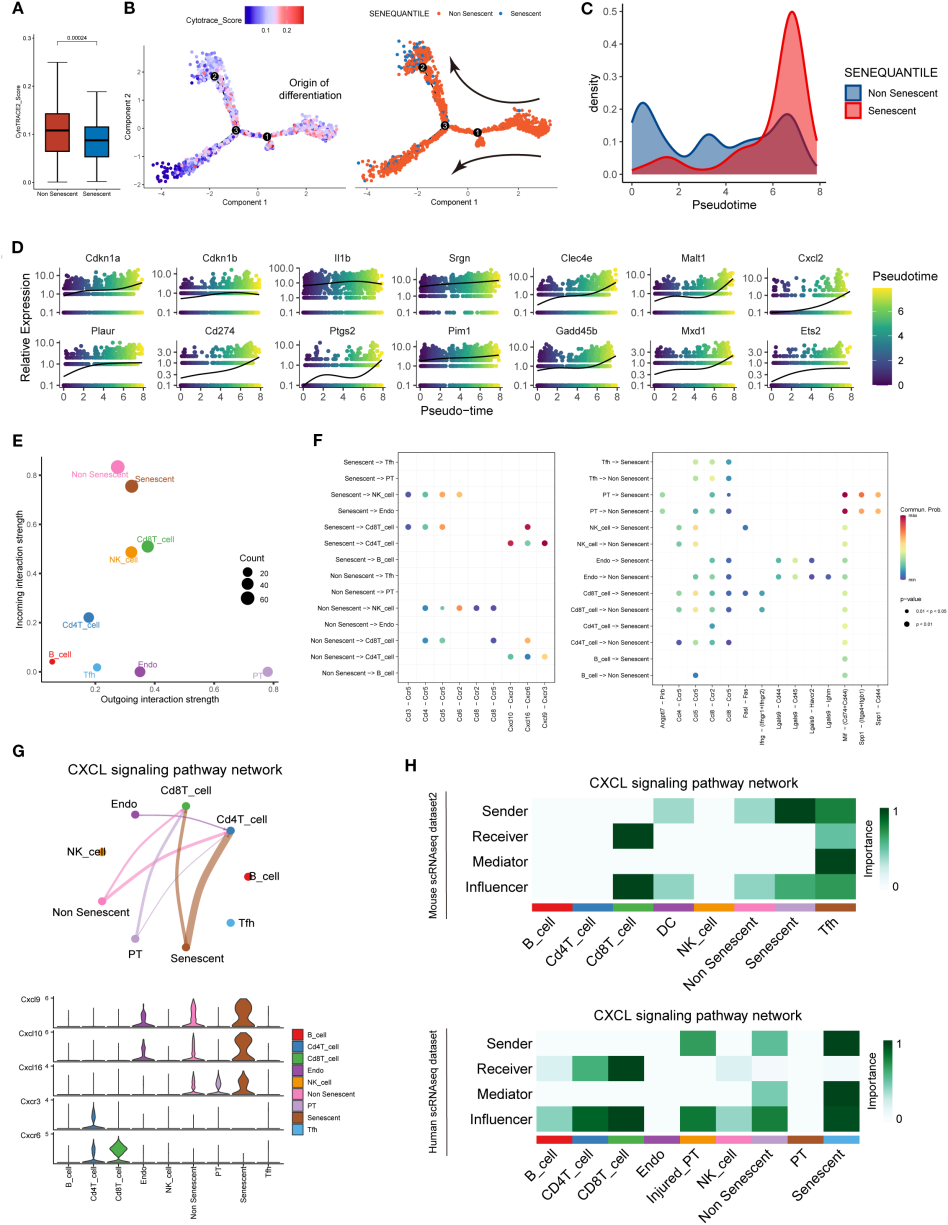
SnIMs accumulation patterns and potential interactive signaling with T cells. **(A)** Boxplots comparing CytoTRACE scores (differentiation potential) between SnIMs and non-senescent IMs (*p* = 0.00024, two sided Wilcox-test). **(B)** Monocle trajectory displaying the origin of differentiation of IMs determined by cytotrace score, and the direction of differentiation from not senescent IMs to SnIMs. **(C)** Density plot demonstrating pseudotime-dependent expansion of SnIM proportion. **(D)** Expression patterns of genes of SnIMs’ signature along pseudotime. **(E)** Dot plot showing the incoming and outcoming interaction strength of cells in alloimmune microenvironment, with the size of dots representing counts of interactions. **(F)** Bubble plots comparing the outgoing communication patterns of senescent and not senescent IMs (left) and the incoming communication patterns of target cells (right). The size of each dot is proportional to the contribution score calculated by pattern recognition analysis. Higher contribution scores indicated richer signaling pathways in the corresponding cell group. **(G)** Circle plot (upper) of CXCL signaling pathway network between cells in alloimmune microenvironment, with the thickness of the line representing strength of interactions. Violin plot (lower) showing the cell-type-specific expression of CXCL ligands (Cxcl9/10/16) and receptors (Cxcr3/6). **(H)** Heatmap showing various signaling functions (sender, receiver, mediator, and influencer) in the Cxcl pathway of alloimmune cells of independent mouse (upper) and human (lower) single-cell databases.

A distinct subpopulation of senescent macrophages within the AME was identified through single-cell transcriptomic profiling. Then, pseudotime trajectory and cell-cell communication analyses were conducted to track their temporal dynamics during acute rejection and their cellular interactions with adjacent immune cells as well as renal parenchymal cells. Significantly diminished differentiation potential was observed in SnIMs through Cytotrace2 analysis (**Figure 6A**). Monocle2 trajectory inference localized SnIMs predominantly at trajectory termini (**Figure 6B**), suggesting progressive accumulation of SnIMs during rejection. This temporal accumulation pattern was further ​supported​ by pseudotime density plots, showing peak SnIM enrichment at pseudotime terminus (**Figure 6C**). Moreover, SnIM signature genes exhibited pseudotime-dependent upregulation in IMs (**Figure 6D**). CellChat analysis showed that SnIMs displayed enhanced incoming interactions with other cells (**Figure 6E**), and tubular epithelial cells served as prominent singling senders to them through Mif-(Cd74+Cd44) and Spp1-(Itga4+Itgb1) ligand-receptor axes (**Figure 6F**). This ​could potentially contribute to​ SnIM recruitment to peritubular areas. Notably, interaction potentials​ were identified between CD4+ T cells and CD8+ T cells through Cxcl9/Cxcl10-Cxcr3 and Cxcl16-Cxcr6 pathways, respectively (**Figures 6F, G**). This observed crosstalk ​suggests a potential mechanism​ by which SnIMs, via sustained SASP secretion (notably Cxcl chemokines), ​might participate in sustaining​ inflammatory responses ​associated with​ rejection injury. ​​Importantly,​​ similar SnIM-T cell interaction patterns were ​replicated​ in independent mouse and human scRNA-seq datasets (**Figure 6H**).

### 3.6 SnIMs abundance shows association with graft outcomes and rejection injury

**Figure 7:**
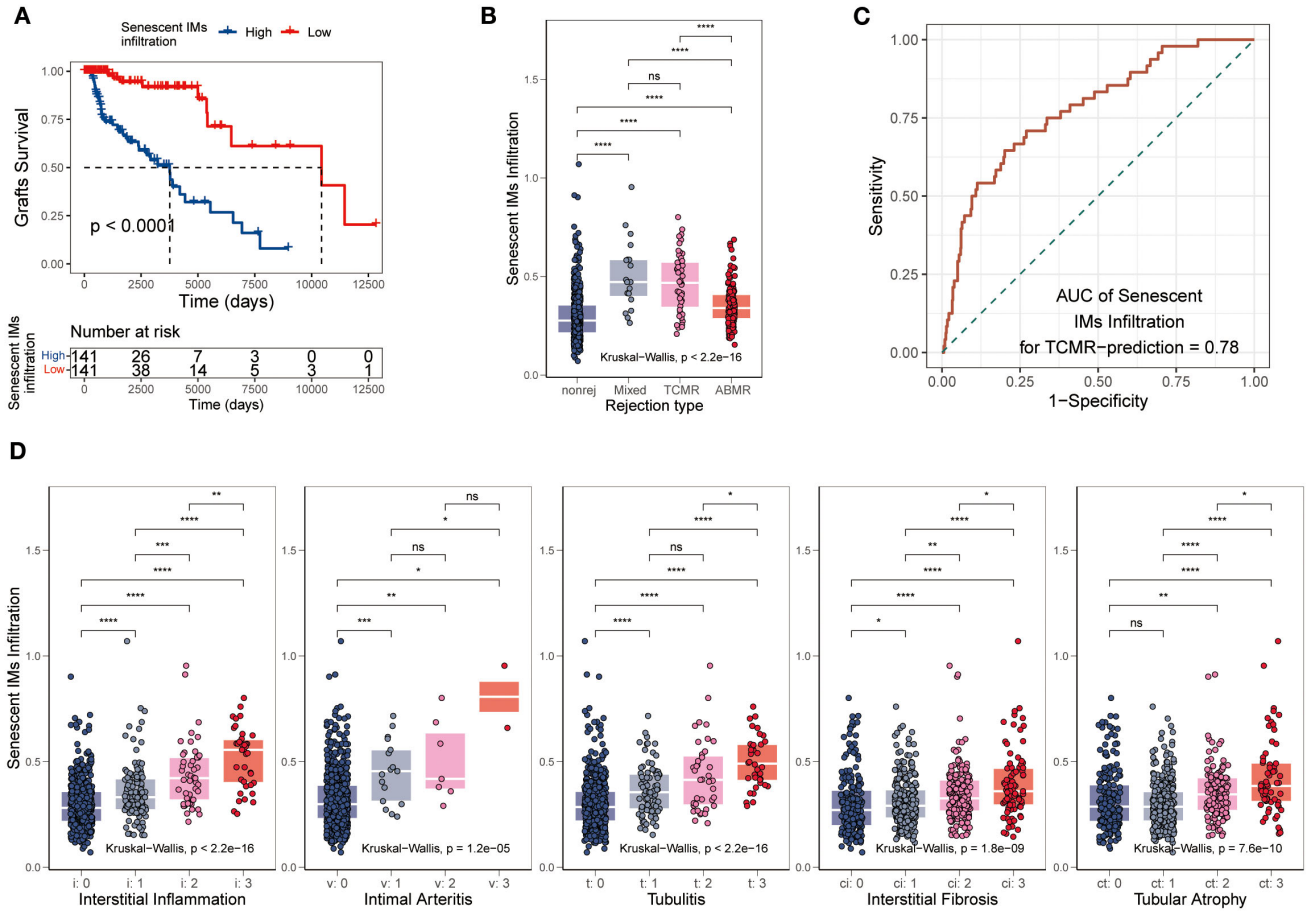
Association of SnIMs infiltration with clinical outcomes. **(A)** Kaplan-Meier curves showing inferior graft survival in patients with high SnIMs infiltration versus low infiltration (*p* < 0.001, log-rank test). **(B)** Boxplot comparing SnIMs infiltration across rejection subtypes: antibody-mediated rejection (AMR), T cell-mediated rejection (TCMR), mixed rejection, and non-rejection controls (*p* < 0.001, Kruskal-Wallis test). **(C)** ROC curves assessing the predictive ability of SnIMs infiltration for TCMR (area under the curve, AUC = 0.78). **(D)** Boxplot comparing SnIMs infiltration across Banff lesion score: interstitial inflammation (i), intimal arteritis (v), tubulitis (t), interstitial fibrosis (ci), tubular atrophy (ct) (*p* < 0.001, Kruskal-Wallis test).

To explore the clinical value of SnIMs, we quantified their infiltration abundance in renal transplant biopsy cohorts using ssGSEA. A correlative relationship was observed, with patients showing high SnIM infiltration levels experiencing poorer graft survival (*p* < 0.0001) (**Figure 7A**). SnIMs enrichment was ​notably higher​ in TCMR and mixed rejection biopsies (**Figure 7B**) and displayed good predictive performance for TCMR diagnosis (AUC = 0.78; **Figure 7C**). Furthermore, SnIMs infiltration showed ​stepwise positive correlations​ with key Banff lesion scores, including interstitial inflammation (i), intimal arteritis (v), tubulitis (t), interstitial fibrosis (ci), and tubular atrophy (ct) (**Figure 7D**). Collectively, these data associate increased SnIMs infiltration with acute/chronic allograft injury and inferior clinical outcomes.​​ ​Based on these correlative findings, targeted depletion of SnIMs represents a hypothesis warranting future investigation to assess its potential for improving long-term graft survival.

## 4 Discussion

CS is multifactorial in organ transplantation, including donor/recipient age, the recipient’s chronic medical conditions, IRI during organ implantation, alloimmune responses against the donor organ, and post-transplant recurrence of primary diseases (8, 33). Up to now, the age-and IRI-related senescence mechanisms are relatively well understood, while our study focuses on the interplay between rejection and CS. First, transcriptomic analysis of renal transplant biopsy specimens revealed that CS is a characteristic phenotype in rejection. Subsequently, dynamic evolution of senescence phenotypes in the AME were found in age-matched rat renal transplant models: alloimmune response acts as secondary stressors that exacerbate the senescence process initiated by IRI, characterized by accelerated senescence of immune cells, particularly macrophages. Single-cell analysis further confirmed the presence of transcriptionally conserved SnIMs in cross-species rejection samples. These cells accumulate as rejection progresses and mediate sustained inflammatory responses via their SASP (predominantly via CXCL chemokine family members). Clinical cohort studies also validated that the infiltration of SnIMs in renal allografts correlates with both Banff lesion severity and adverse transplant outcomes.

A recent study found that transplantation-stress induces CS in age-matched corneal grafts and targeted elimination of senescent cells could effectively reduce rejection immune reaction. While the authors observed accelerated senescence phenotypes in allogeneic grafts compared to syngeneic controls (primarily in CD45+ immune cells, aligning with our findings), they didn’t elucidate the source of stress only attributing senescence to general “transplantation injury” (34). Based on existing evidence, we speculate that allograft rejection may serve as a key trigger for cellular senescence: alloantigens excessively activate immune response and generate robust inflammatory cascades. Notably, a bidirectional interplay exists between inflammation and senescence: on one hand, hyperactive inflammation predisposes cells to SIPS; on the other, senescent cells induce paracrine senescence via the SASP, creating a self-amplifying “inflammation-senescence” vicious cycle (5, 35). Moreover, damage-associated molecular patterns (DAMPs) likely act as central hubs in this crosstalk. In solid organ transplantation, initial DAMP release triggered by IRI activates innate immunity and subsequent alloimmune responses inflict secondary damage on graft cells, perpetuating DAMP generation (36, 37). As critical mediators bridging innate and adaptive immunity, DAMPs establish a positive feedback loop through three interconnected pathways: 1. DAMP-TLR signaling activates cell cycle arrest pathways and SASP secretion (38, 39); 2. SASP components stimulate further DAMP release (40); 3. Sustained DAMPs exacerbate the inflammatory response and potentiate rejection (37). This “DAMP-senescence-DAMP” amplification loop may provide a novel explanatory framework for post-transplant rejection pathogenesis.

As central components of the innate immune system, macrophages initiate adaptive immune responses through TLR-mediated recognition of DAMPs released from injured grafts (41). Notably, this biological predisposition renders macrophages vulnerable to the “DAMP-senescence” vicious cycle, establishing them as primary effectors of cellular senescence in acute organ injury. Marked macrophage senescence was found in CCl4-induced liver injury models and it in return aggravated liver tissue damage (42). Similarly, our study identifies IMs as predominant senescent immune cell populations in allograft rejection. Currently, the absence of single, universal, or specific biomarkers for identifying senescent immune cells poses significant challenges to senescence research *in vivo (* 43). We successfully identified *Cdkn1a*+SnIMs in rejecting renal grafts and delineated their conserved transcriptomic gene-expression profiles across species by analyzing singe-cell datasets. While cell cycle inhibitors p16 and p21 are conventionally employed as senescence markers, our selection of *Cdkn1a* (p21) as the principal biomarker is methodologically justified: 1. Technical limitations in sequencing platforms result in approximately 50% dropout rates for *Cdkn2a* (p16) transcripts (44); 2. p21 has been proved to be more related to SIPS while p16 is mainly involved in regulating replicative senescence (45, 46). Recent studies have demonstrated p21 upregulation coupled with rapid senescence emergence following hemorrhagic shock (47). Although *Cdkn1a*+SnIMs exhibits limited potential for differentiation as validated by Cytotrace, they achieve persistent survival through the following dual mechanisms: 1. Dysregulated activation of anti-apoptotic pathways (e.g., BCL-2 family); 2. immune evasion mediated by overexpressed immune checkpoint molecule CD274 (PD-L1). This survival paradigm explains their progressive accumulation during rejection and suggests enduring graft-destructive effects. In renal transplant biopsies, SnIM infiltration correlates not only with acute injury (interstitial inflammation, endarteritis, tubulitis) but also chronic allograft lesions (interstitial fibrosis/tubular atrophy, IF/TA). Pre-transplant treatment of aged donor kidneys with BCL-2 inhibitor ABT-263 removes senescent cells by inducing apoptosis and significantly improves functional recovery after transplantation (9). Given that, we have good reasons to see ABT-263-based senolytic therapy as a promising strategy for eliminating senescent macrophages to mitigate rejection-mediated injury post- transplantation.

The SASP, a hallmark of cellular senescence, perpetuates a low-grade inflammatory microenvironment through secretion of proinflammatory cytokines and chemokines, mediating paracrine propagation of senescent phenotypes and triggering secondary cell death (48). Single-cell analysis revealed that SnIMs exhibit specific overexpression of canonical SASP component genes, including *Il1b, Cxcl2*, and *Cxcl10*. The transcription factor NF-κB occupies a central regulatory role within the SASP network. Protein kinase D (PKD) and CCAAT/enhancer-binding protein β (C/EBPβ) cooperatively interact with NF-κB to activate SASP gene transcription (49). Our analysis surmised NF-κB family members (Nfkb1, Rela, Ikbkb) and Cebpb as key TFs predicted to govern the gene networks of senescent IMs. Intercellular communication analysis further uncovered that CXCL chemokine family members (Cxcl9, Cxcl10, Cxcl16) constitute the principal molecular that connecting SnIMs and effector T cells. Notably, CXCL9/CXCL10 in blood and grafts is widely recognized as a biomarker of rejection, due to their biological function of T cell recruitment (50). Similarly, CXCL16 demonstrates T cell chemotactic activity within the inflammatory milieu of renal allografts (51). SnIMs’ persistent chemoattraction of effector T cells is likely the key factor for the acute and chronic graft injury. This may partly explain SnIMs’ more pronounced infiltration in TCMR and could be used as a biomarker to predict TCMR.

We acknowledge several limitations in our study. Firstly, while rejection stress-induced cellular senescence was observed in age-matched rat renal transplant models and a “DAMP-senescence-DAMP” feedback loop hypothesis was proposed to rationalize the findings, the underlying mechanisms and potential differences in senescence induction pathways of TCMR and AMR require deeper exploration. Secondly, although single-cell analyses inferred interactions between senescent macrophages and T cells, co-culture experiments are needed to dissect direct cellular crosstalk between senescent cells and their effector targets, thereby explaining their enhanced immunopathogenic functions. Finally, the CS gene sets utilized herein - in particular, the SenMayo signature - as well as our proposed SnIM signature, incorporate numerous inflammatory components of the SASP. As these genes are also induced during macrophage activation, their upregulation may not exclusively reflect senescence, potentially leading to false-positive interpretations in the context of immune responses. However, macrophage activation and CS are not mutually exclusive biological states. Recent evidence suggests that inflammatory stimuli (e.g., LPS) can concurrently induce both macrophage activation and senescence (52). Future studies should focus on identifying more specific markers that can unequivocally distinguish senescent macrophages, particularly in inflammatory microenvironments.

In summary, our findings shed light on the role of CS in renal allograft rejection. We successfully identified and characterized novel senescent macrophages induced by rejection stress in silico and *in vivo*. The observed accumulation of SnIMs ​appears linked​ to graft injury​ and poor outcomes. ​These findings position​ SnIMs ​as a candidate biomarker​ for rejection severity ​and suggest their potential as a therapeutic candidate worthy of future experimental validation.

## Data Availability

The original contributions presented in the study are included in the article/**Supplementary Material**. Further inquiries can be directed to the corresponding authors.

## References

[1] Aubert O Ursule-Dufait C Brousse R Gueguen J Racape M Raynaud M. Cell-free DNA for the detection of kidney allograft rejection. Nat Med. (2024) 30:2320–7. doi: 10.1038/s41591-024-03087-3, PMID: 38824959 PMC11333280

[2] Muduma G Odeyemi I Smith-Palmer J Pollock RF. Review of the clinical and economic burden of antibody-mediated rejection in renal transplant recipients. Adv Ther. (2016) 33:345–56. doi: 10.1007/s12325-016-0292-y, PMID: 26905265

[3] Cucchiari D Podesta MA Ponticelli C. Pathophysiology of rejection in kidney transplantation. Expert Rev Clin Immunol. (2024) 20:1471–81. doi: 10.1080/1744666X.2024.2421310, PMID: 39467249

[4] Huang W Hickson LJ Eirin A Kirkland JL Lerman LO. Cellular senescence: the good, the bad and the unknown. Nat Rev Nephrol. (2022) 18:611–27. doi: 10.1038/s41581-022-00601-z, PMID: 35922662 PMC9362342

[5] Chen C Zheng M Hou H Fang S Chen L Yang J. Cellular senescence in ischemia/reperfusion injury. Cell Death Discov. (2022) 8:420. doi: 10.1038/s41420-022-01205-z, PMID: 36253355 PMC9576687

[6] Sis B Tasanarong A Khoshjou F Dadras F Solez K Halloran PF. Accelerated expression of senescence associated cell cycle inhibitor p16INK4A in kidneys with glomerular disease. Kidney Int. (2007) 71:218–26. doi: 10.1038/sj.ki.5002039, PMID: 17183247

[7] McGlynn LM Stevenson K Lamb K Zino S Brown M Prina A. Cellular senescence in pretransplant renal biopsies predicts postoperative organ function. Aging Cell. (2009) 8:45–51. doi: 10.1111/j.1474-9726.2008.00447.x, PMID: 19067655

[8] Kirchner VA Badshah JS Hong SK Martinez O Pruett TL Niedernhofer LJ. Effect of cellular senescence in disease progression and transplantation: immune cells and solid organs. TRANSPLANTATION. (2024) 108:1509–23. doi: 10.1097/TP.0000000000004838, PMID: 37953486 PMC11089077

[9] He A Sarwar A Thole L Siegle J Sattler A Ashraf MI. Renal inflamm-aging provokes intra-graft inflammation following experimental kidney transplantation. Am J Transplant. (2022) 22:2529–47. doi: 10.1111/ajt.17154, PMID: 35851547

[10] van Willigenburg H de Keizer P de Bruin R. Cellular senescence as a therapeutic target to improve renal transplantation outcome. Pharmacol Res. (2018) 130:322–30. doi: 10.1016/j.phrs.2018.02.015, PMID: 29471104

[11] Chkhotua AB Gabusi E Altimari A D’Errico A Yakubovich M Vienken J. Increased expression of p16(INK4a) and p27(Kip1) cyclin-dependent kinase inhibitor genes in aging human kidney and chronic allograft nephropathy. Am J Kidney Dis. (2003) 41:1303–13. doi: 10.1016/S0272-6386(03)00363-9, PMID: 12776284

[12] Yousefzadeh MJ Flores RR Zhu Y Schmiechen ZC Brooks RW Trussoni CE. An aged immune system drives senescence and ageing of solid organs. NATURE. (2021) 594:100–5. doi: 10.1038/s41586-021-03547-7, PMID: 33981041 PMC8684299

[13] Suryadevara V Hudgins AD Rajesh A Pappalardo A Karpova A Dey AK. SenNet recommendations for detecting senescent cells in different tissues. Nat Rev Mol Cell Biol. (2024) 25:1001–23. doi: 10.1038/s41580-024-00738-8, PMID: 38831121 PMC11578798

[14] Einecke G Reeve J Sis B Mengel M Hidalgo L Famulski KS. A molecular classifier for predicting future graft loss in late kidney transplant biopsies. J Clin Invest. (2010) 120:1862–72. doi: 10.1172/JCI41789, PMID: 20501945 PMC2877953

[15] Yokose T Szuter ES Rosales I Guinn MT Liss AS Baba T. Dysfunction of infiltrating cytotoxic CD8+ T cells within the graft promotes murine kidney allotransplant tolerance. J Clin Invest. (2024) 134(16):e179709. doi: 10.1172/JCI179709, PMID: 38888968 PMC11324304

[16] Dangi A Natesh NR Husain I Ji Z Barisoni L Kwun J. Single cell transcriptomics of mouse kidney transplants reveals a myeloid cell pathway for transplant rejection. JCI Insight. (2020) 5(20):e141321. doi: 10.1172/jci.insight.141321, PMID: 32970632 PMC7605544

[17] Leckie-Harre A Silverman I Wu H Humphreys BD Malone AF. Sequencing of physically interacting cells in human kidney allograft rejection to infer contact-dependent immune cell transcription. TRANSPLANTATION. (2024) 108:421–9. doi: 10.1097/TP.0000000000004762, PMID: 37638864 PMC10798591

[18] John SV Seim GL Erazo-Flores BJ Votava JA Urquiza US Arp NL. Classically activated macrophages undergo functionally significant nucleotide metabolism remodelling driven by nitric oxide. Nat Metab. (2025) 7:1681–702. doi: 10.1038/s42255-025-01337-3, PMID: 40759751 PMC12356500

[19] Subramanian A Tamayo P Mootha VK Mukherjee S Ebert BL Gillette MA. Gene set enrichment analysis: a knowledge-based approach for interpreting genome-wide expression profiles. Proc Natl Acad Sci U.S.A. (2005) 102:15545–50. doi: 10.1073/pnas.0506580102, PMID: 16199517 PMC1239896

[20] Hanzelmann S Castelo R Guinney J. GSVA: gene set variation analysis for microarray and RNA-seq data. BMC Bioinf. (2013) 14:7. doi: 10.1186/1471-2105-14-7, PMID: 23323831 PMC3618321

[21] Lu X Meng J Su L Jiang L Wang H Zhu J. Multi-omics consensus ensemble refines the classification of muscle-invasive bladder cancer with stratified prognosis, tumour microenvironment and distinct sensitivity to frontline therapies. Clin Transl Med. (2021) 11:e601. doi: 10.1002/ctm2.601, PMID: 34936229 PMC8693439

[22] Korsunsky I Millard N Fan J Slowikowski K Zhang F Wei K. Fast, sensitive and accurate integration of single-cell data with Harmony. Nat Methods. (2019) 16:1289–96. doi: 10.1038/s41592-019-0619-0, PMID: 31740819 PMC6884693

[23] Hu C Li T Xu Y Zhang X Li F Bai J. CellMarker 2.0: an updated database of manually curated cell markers in human/mouse and web tools based on scRNA-seq data. Nucleic Acids Res. (2023) 51:D870–6. doi: 10.1093/nar/gkac947, PMID: 36300619 PMC9825416

[24] Saul D Kosinsky RL Atkinson EJ Doolittle ML Zhang X LeBrasseur NK. A new gene set identifies senescent cells and predicts senescence-associated pathways across tissues. Nat Commun. (2022) 13:4827. doi: 10.1038/s41467-022-32552-1, PMID: 35974106 PMC9381717

[25] Troiani M Colucci M D’Ambrosio M Guccini I Pasquini E Varesi A. Single-cell transcriptomics identifies Mcl-1 as a target for senolytic therapy in cancer. Nat Commun. (2022) 13:2177. doi: 10.1038/s41467-022-29824-1, PMID: 35449130 PMC9023465

[26] Zhou Y Zhou B Pache L Chang M Khodabakhshi AH Tanaseichuk O. Metascape provides a biologist-oriented resource for the analysis of systems-level datasets. Nat Commun. (2019) 10:1523. doi: 10.1038/s41467-019-09234-6, PMID: 30944313 PMC6447622

[27] Qiu X Mao Q Tang Y Wang L Chawla R Pliner HA. Reversed graph embedding resolves complex single-cell trajectories. Nat Methods. (2017) 14:979–82. doi: 10.1038/nmeth.4402, PMID: 28825705 PMC5764547

[28] Gulati GS Sikandar SS Wesche DJ Manjunath A Bharadwaj A Berger MJ. Single-cell transcriptional diversity is a hallmark of developmental potential. SCIENCE. (2020) 367:405–11. doi: 10.1126/science.aax0249, PMID: 31974247 PMC7694873

[29] Jin S Plikus MV Nie Q. CellChat for systematic analysis of cell-cell communication from single-cell transcriptomics. Nat Protoc. (2025) 20:180–219. doi: 10.1038/s41596-024-01045-4, PMID: 39289562

[30] Mengel M Loupy A Haas M Roufosse C Naesens M Akalin E. Banff 2019 Meeting Report: Molecular diagnostics in solid organ transplantation-Consensus for the Banff Human Organ Transplant (B-HOT) gene panel and open source multicenter validation. Am J Transplant. (2020) 20:2305–17. doi: 10.1111/ajt.16059, PMID: 32428337 PMC7496585

[31] Robertson H Kim HJ Li J Robertson N Robertson P Jimenez-Vera E. Decoding the hallmarks of allograft dysfunction with a comprehensive pan-organ transcriptomic atlas. Nat Med. (2024) 30:3748–57. doi: 10.1038/s41591-024-03030-6, PMID: 38890530 PMC11645273

[32] Amor C Feucht J Leibold J Ho YJ Zhu C Alonso-Curbelo D. Senolytic CAR T cells reverse senescence-associated pathologies. NATURE. (2020) 583:127–32. doi: 10.1038/s41586-020-2403-9, PMID: 32555459 PMC7583560

[33] Iske J Seyda M Heinbokel T Maenosono R Minami K Nian Y. Senolytics prevent mt-DNA-induced inflammation and promote the survival of aged organs following transplantation. Nat Commun. (2020) 11:4289. doi: 10.1038/s41467-020-18039-x, PMID: 32855397 PMC7453018

[34] Chi H Ma L Zeng F Wang X Peng P Bai X. Senolytic treatment alleviates corneal allograft rejection through upregulation of angiotensin-converting enzyme 2 (ACE2). Invest Ophthalmol Vis Sci. (2025) 66:15. doi: 10.1167/iovs.66.2.15, PMID: 39913165 PMC11806429

[35] Jin H Zhang Y Ding Q Wang SS Rastogi P Dai DF. Epithelial innate immunity mediates tubular cell senescence after kidney injury. JCI Insight. (2019) 4(2):e125490. doi: 10.1172/jci.insight.125490, PMID: 30674725 PMC6413792

[36] Land WG Agostinis P Gasser S Garg AD Linkermann A. Transplantation and damage-associated molecular patterns (DAMPs). Am J Transplant. (2016) 16:3338–61. doi: 10.1111/ajt.13963, PMID: 27421829

[37] Todd JL Palmer SM. Danger signals in regulating the immune response to solid organ transplantation. J Clin Invest. (2017) 127:2464–72. doi: 10.1172/JCI90594, PMID: 28530643 PMC5490743

[38] Hari P Millar FR Tarrats N Birch J Quintanilla A Rink CJ. The innate immune sensor Toll-like receptor 2 controls the senescence-associated secretory phenotype. Sci Adv. (2019) 5:w254. doi: 10.1126/sciadv.aaw0254, PMID: 31183403 PMC6551188

[39] Mannarino M Cherif H Li L Sheng K Rabau O Jarzem P. Toll-like receptor 2 induced senescence in intervertebral disc cells of patients with back pain can be attenuated by o-vanillin. Arthritis Res Ther. (2021) 23:117. doi: 10.1186/s13075-021-02504-z, PMID: 33863359 PMC8051055

[40] Chen F Tang H Cai X Lin J Kang R Tang D. DAMPs in immunosenescence and cancer. Semin Cancer Biol. (2024) 106-107:123–42. doi: 10.1016/j.semcancer.2024.09.005, PMID: 39349230

[41] Braza F Brouard S Chadban S Goldstein DR. Role of TLRs and DAMPs in allograft inflammation and transplant outcomes. Nat Rev Nephrol. (2016) 12:281–90. doi: 10.1038/nrneph.2016.41, PMID: 27026348 PMC7952035

[42] Zhao H Liu Z Chen H Han M Zhang M Liu K. Identifying specific functional roles for senescence across cell types. CELL. (2024) 187:7314–34. doi: 10.1016/j.cell.2024.09.021, PMID: 39368477

[43] Sharpless NE Sherr CJ. Forging a signature of *in vivo* senescence. Nat Rev Cancer. (2015) 15:397–408. doi: 10.1038/nrc3960, PMID: 26105537

[44] Faggioli F Velarde MC Wiley CD. Cellular senescence, a novel area of investigation for metastatic diseases. CELLS-BASEL. (2023) 12(6):860. doi: 10.3390/cells12060860, PMID: 36980201 PMC10047218

[45] Luo Y Zou P Zou J Wang J Zhou D Liu L. Autophagy regulates ROS-induced cellular senescence via p21 in a p38 MAPKalpha dependent manner. Exp GERONTOL. (2011) 46:860–7. doi: 10.1016/j.exger.2011.07.005, PMID: 21816217 PMC3390188

[46] Mirzayans R Andrais B Hansen G Murray D. Role of p16(INK4A) in Replicative Senescence and DNA Damage-Induced Premature Senescence in p53-Deficient Human Cells. Biochem Res Int. (2012) 2012:951574. doi: 10.1155/2012/951574, PMID: 22924132 PMC3424640

[47] Chu X Wen J Raju RP. Rapid senescence-like response after acute injury. Aging Cell. (2020) 19:e13201. doi: 10.1111/acel.13201, PMID: 32741083 PMC7511876

[48] Wang B Han J Elisseeff JH Demaria M. The senescence-associated secretory phenotype and its physiological and pathological implications. Nat Rev Mol Cell Biol. (2024) 25:958–78. doi: 10.1038/s41580-024-00727-x, PMID: 38654098

[49] Lopes-Paciencia S Saint-Germain E Rowell MC Ruiz AF Kalegari P Ferbeyre G. The senescence-associated secretory phenotype and its regulation. CYTOKINE. (2019) 117:15–22. doi: 10.1016/j.cyto.2019.01.013, PMID: 30776684

[50] Zhuang J Shan Z Ma T Li C Qiu S Zhou X. CXCL9 and CXCL10 accelerate acute transplant rejection mediated by alloreactive memory T cells in a mouse retransplantation model. Exp Ther Med. (2014) 8:237–42. doi: 10.3892/etm.2014.1714, PMID: 24944628 PMC4061216

[51] Schramme A Abdel-Bakky MS Gutwein P Obermuller N Baer PC Hauser IA. Characterization of CXCL16 and ADAM10 in the normal and transplanted kidney. Kidney Int. (2008) 74:328–38. doi: 10.1038/ki.2008.181, PMID: 18480749

[52] Wang H Fu H Zhu R Wu X Ji X Li X. BRD4 contributes to LPS-induced macrophage senescence and promotes progression of atherosclerosis-associated lipid uptake. Aging (Albany NY). (2020) 12:9240–59. doi: 10.18632/aging.103200, PMID: 32392533 PMC7288959

[53] Reeve J Bohmig GA Eskandary F Einecke G Lefaucheur C Loupy A. Assessing rejection-related disease in kidney transplant biopsies based on archetypal analysis of molecular phenotypes. JCI Insight. (2017) 2(12):e94197. doi: 10.1172/jci.insight.94197, PMID: 28614805 PMC5470931

