# Supplementary material for: Identification and characterization of senescent macrophages in renal allograft rejection: a cross-species MultiOmics study
Source: Front Immunol. 2025 Oct 9;16:1623124. doi: 10.3389/fimmu.2025.1623124 (PMC12545005; doi:10.3389/fimmu.2025.1623124)
Supplement: Supplementary file 1 [file Supplementaryfile1.docx]

## **SUPPLEMENTARY 1**

## **Table S1** **Characteristic at cohort and biopsy.**

| **GEO accession** | **GSE21374** | **GSE98320** |
| --- | --- | --- |
| **Cohort type in study** | Prognosis cohort | Banff cohort |
| **Platform** | GPL570 | GPL15207 |
| **Sample tissue** | kidney transplant biopsies | kidney transplant biopsies |
| **Sample size** | 105/282* | 1208 |
| **Number of patients** | 105 | 1045 |
| **Indication for biopsy** | | |
| Primary nonfunction (including DGF) | unknown | 53 (5%) |
| Deterioration of graft function | 65 (62%) | 436 (36%) |
| Stable impaired graft function | 7 (7%) | 79 (7%) |
| Investigate proteinuria/rejection/BK/creatinine | 15 (14%) | 175 (14%) |
| Follow-up from previous biopsy unknown 6 | 6 (6%) | unknown |
| Others | 6 (6%) | 443 (37%) |
| Indication unknown | 6 (6%) | 22 (2%) |
| **Time of biopsy after transplant (d)** | | |
| mean time | 1734 | unknown |
| median time (range) | unknown | 591 (1-11453) |
| Early biopsies (< 1 year) | 0 (0%) | 507 (42%) |
| Late biopsies (≥ 1 year) | 100 (100%) | 701 (58%) |
| **Diagnosis (conclusive)** | | |
| TCMR | 14 (13%) | 87 (7%) |
| ABMR | 11 (10%) | 24 (2%) |
| Mixed ABMR and TCMR | 3 (3%) | 41 (3%) |
| Borderline rejection | 11 (10%) | 109 (9%) |
| Transplant glomerulopathy (TG) | unknown | 40 (3%) |
| Glomerulonephritis | 22 (21%) | 97 (8%) |
| BK virus | 1 (1%) | 37 (3%) |
| No major abnormalities | unknown | 274 (23%) |
| **Maintenance immunosuppression at biopsy (calcineurin inhibitors)** | | |
| Tacrolimus | 38 (36%) | 712 (59%) |
| Cyclosporine | 49 (47%) | 192 (16%) |
| **Time of follow-up after biopsy (d, mean time)** | 774 | unknown |
| **Failed grafts** | 30 (29%) | unknown |

*GSE21374 provided a total of 282 samples, but was only able to find histological information for 105 of them.

## **Table S2 Information on the cellular senescence gene sets used in the study**

| **Gene set/signature name** | **Exact source** | **Link** |
| --- | --- | --- |
| REACTOME: CELLULAR SENESCENCE | R-HSA-2559583 | https://www.reactome.org/content/detail/R-HSA-2559583 |
| GOBP: CELL CYCLE ARREST | GO:0007050 | http://amigo.geneontology.org/amigo/term/GO:0007050 |
| GOBP: STRESS INDUCED PREMATURE SENESCENCE | GO:0090400 | http://amigo.geneontology.org/amigo/term/GO:0090400 |
| REACTOME: SENESCENCE- ASSOCIATED SECRETORY PHENOTYPE_SASP | R-MMU-2559582 | https://www.reactome.org/content/detail/R-MMU-2559582 |
| SenMayo signature | - | https://www.nature.com/articles/s41467-022-32552-1 |
| Senescence signature |  | https://www.nature.com/articles/s41467-022-29824-1 |

## **Table S3 Gene lists on the cellular senescence gene sets used in the study**

| REACTOME: CELLULAR SENESCENCE | GOBP: CELL CYCLE ARREST | GOBP: STRESS INDUCED PREMATURE SENESCENCE | REACTOME: SENESCENCE- ASSOCIATED SECRETORY PHENOTYPE_SASP | SenMayo signature | Senescence signature |
| --- | --- | --- | --- | --- | --- |
| ACD  AGO1  AGO3  AGO4  ANAPC1  ANAPC10  ANAPC11  ANAPC15  ANAPC16  ANAPC2  ANAPC4  ANAPC5  ANAPC7  ASF1A  ATM  BMI1  CABIN1  CBX2  CBX4  CBX6  CBX8  CCNA1  CCNA2  CCNE1  CCNE2  CDC16  CDC23  CDC26  CDC27  CDK2  CDK4  CDK6  CDKN1A  CDKN1B  CDKN2A  CDKN2B  CDKN2C  CDKN2D  CEBPB  CXCL8  E2F1  E2F2  E2F3  EED  EHMT1  EHMT2  EP400  ERF  ETS1  ETS2  EZH2  FOS  FZR1  H1-0  H1-1  H1-2  H1-3  H1-4  H1-5  H2AB1  H2AC14  H2AC18  H2AC19  H2AC20  H2AC4  H2AC6  H2AC7  H2AC8  H2AJ  H2AX  H2AZ2  H2BC1  H2BC10  H2BC11  H2BC12  H2BC12L  H2BC13  H2BC14  H2BC15  H2BC17  H2BC21  H2BC26  H2BC3  H2BC4  H2BC5  H2BC6  H2BC7  H2BC8  H2BC9  H3-3A  H3-3B  H3-4  H3C1  H3C10  H3C11  H3C12  H3C13  H3C14  H3C15  H3C2  H3C3  H3C4  H3C6  H3C7  H3C8  H4C1  H4C11  H4C12  H4C13  H4C14  H4C15  H4C16  H4C2  H4C3  H4C4  H4C5  H4C6  H4C8  H4C9  HIRA  HMGA1  HMGA2  ID1  IFNB1  IGFBP7  IL1A  IL6  JUN  KAT5  KDM6B  LMNB1  MAP2K3  MAP2K4  MAP2K6  MAP2K7  MAP3K5  MAP4K4  MAPK1  MAPK10  MAPK11  MAPK14  MAPK3  MAPK7  MAPK8  MAPK9  MAPKAPK2  MAPKAPK3  MAPKAPK5  MDM2  MDM4  MINK1  MIR24-1  MIR24-2  MOV10  MRE11  NBN  NFKB1  PHC1  PHC2  PHC3  POT1  RAD50  RB1  RBBP4  RBBP7  RELA  RING1  RNF2  RPS27A  RPS6KA1  RPS6KA2  RPS6KA3  SCMH1  SP1  STAT3  SUZ12  TERF1  TERF2  TERF2IP  TFDP1  TFDP2  TINF2  TNIK  TNRC6A  TNRC6B  TNRC6C  TP53  TXN  UBA52  UBB  UBC  UBE2C  UBE2D1  UBE2E1  UBE2S  UBN1  VENTX | ABL1  ADAM10  AKT2  APBB1  APBB2  APC  ARID3A  ATM  AURKA  BARD1  BAX  BIN1  BRCA1  BRINP1  BRINP2  BRINP3  BTG2  BTG4  CAB39  CAB39L  CALR  CARM1  CASP2  CCNB1  CCND1  CDC123  CDC14A  CDC14B  CDC14C  CDC25C  CDK1  CDK2  CDK4  CDK5  CDK5R1  CDK6  CDK7  CDK9  CDKN1A  CDKN1B  CDKN1C  CDKN2A  CDKN2B  CDKN2C  CDKN2D  CDKN3  CGREF1  CGRRF1  CHEK2  CNOT1  CNOT10  CNOT11  CNOT2  CNOT3  CNOT4  CNOT6  CNOT6L  CNOT7  CNOT8  CNOT9  CRADD  CRLF3  CXCL8  DAB2IP  DDIAS  DDIT3  DHCR24  DUSP1  E2F1  E2F4  E2F7  E2F8  E4F1  EIF2AK4  EIF4G2  EP300  ERN1  ERN2  EVI2B  FAP  FGF10  FOXE3  FOXM1  FOXO4  FZD9  GADD45A  GADD45GIP1  GAS1  GAS2  GAS2L1  GATA6  GML  GPER1  GTSE1  HBP1  HEPACAM  HMGA2  HMGN5  HRAS  HSP90AB1  ID2  IFNG  IFNW1  IL12A  IL12B  ING4  INHA  INHBA  INSM1  IRF1  IRF6  JMY  KAT2B  KIF20B  KLLN  KMT2E  LAMTOR1  LAMTOR2  LAMTOR3  LAMTOR4  LAMTOR5  MAGI2  MAP2K1  MAP2K6  MAP3K20  MAPK12  MCPH1  MDM2  MDM4  MED25  MIF  MIR152  MIR200B  MIR34A  MLF1  MLST8  MLXIPL  MSH2  MTBP  MTOR  MUC1  MYBBP1A  MYC  MYOG  NBN  NEUROD1  NKX3-1  NOTCH1  NOTCH2  NUPR2  PCBP4  PCNA  PHOX2B  PIDD1  PKD1  PKD2  PLAGL1  PLK2  PLK3  PML  PNPT1  POU4F1  PPM1A  PPM1G  PPP1R15A  PPP1R9B  PPP2R3B  PPP2R5B  PRKAA1  PRKAA2  PRKAB1  PRKAB2  PRKACA  PRKAG1  PRKAG2  PRKAG3  PRMT1  PRNP  PRR11  RASSF1  RB1  RBL2  RGCC  RHEB  RNF112  RPL23  RPL26  RPRM  RPTOR  RRAGA  RRAGB  RRAGC  RRAGD  RRP8  SDE2  SETMAR  SFN  SGSM3  SKIL  SLC25A33  SLC38A9  SMAD3  SOX2  SOX4  STK11  STRADA  STRADB  TBRG1  TBRG4  TCF7L2  TFAP4  TFDP1  TFDP2  TGFB1  TGFB2  TGFBR1  THBS1  TNKS1BP1  TP53  TP53INP1  TP73  TRIAP1  TSG101  UHMK1  UHRF2  VASH1  WDR6  WHAMM  WHAMMP3  WNT10B  ZBTB17  ZBTB49  ZFHX3  ZNF268  ZNF385A | Bmal1  Mapk14  Mapkapk5  Pla2r1  Sirt1  Trp53  Wnt16 | Anapc1  Anapc10  Anapc11  Anapc15  Anapc16  Anapc2  Anapc4  Anapc5  Anapc7  Ccna1  Ccna2  Cdc16  Cdc23  Cdc26  Cdc27  Cdk2  Cdk4  Cdk6  Cdkn1a  Cdkn1b  Cdkn1c  Cdkn2b  Cebpb  Ehmt1  Ehmt2  Fzr1  Mapk1  Mapk3  Mapk7  Rps27a  Rps6ka1  Rps6ka2  Rps6ka3  Uba52  Uba52rt  Ubb  Ubc  Ube2c  Ube2d1  Ube2e1  Ube2s | ACVR1B  ANG  ANGPT1  ANGPTL4  AREG  AXL  BEX3  BMP2  BMP6  C3  CCL1  CCL13  CCL16  CCL2  CCL20  CCL24  CCL26  CCL3  CCL3L1  CCL4  CCL5  CCL7  CCL8  CD55  CD9  CSF1  CSF2  CSF2RB  CST4  CTNNB1  CTSB  CXCL1  CXCL10  CXCL12  CXCL16  CXCL2  CXCL3  CXCL8  CXCR2  DKK1  EDN1  EGF  EGFR  EREG  ESM1  ETS2  FAS  FGF1  FGF2  FGF7  GDF15  GEM  GMFG  HGF  HMGB1  ICAM1  ICAM3  IGF1  IGFBP1  IGFBP2  IGFBP3  IGFBP4  IGFBP5  IGFBP6  IGFBP7  IL10  IL13  IL15  IL18  IL1A  IL1B  IL2  IL32  IL6  IL6ST  IL7  INHA  IQGAP2  ITGA2  ITPKA  JUN  KITLG  LCP1  MIF  MMP1  MMP10  MMP12  MMP13  MMP14  MMP2  MMP3  MMP9  NAP1L4  NRG1  PAPPA  PECAM1  PGF  PIGF  PLAT  PLAU  PLAUR  PTBP1  PTGER2  PTGES  RPS6KA5  SCAMP4  SELPLG  SEMA3F  SERPINB4  SERPINE1  SERPINE2  SPP1  SPX  TIMP2  TNF  TNFRSF10C  TNFRSF11B  TNFRSF1A  TNFRSF1B  TUBGCP2  VEGFA  VEGFC  VGF  WNT16  WNT2 | Cdkn2a  Cdkn1a  Serpine1  Cdkn1b  Cdkn2d  Cdkn2b |

Note: The genes marked in blue are present in the Banff Human Organ Transplant (B‐HOT) gene panel.

## **Table S4 Information on the senescent macrophage transcriptional signatures**

| **Signature Genes** | **Description** | **PMCID of Supporting Literature** |
| --- | --- | --- |
| Cdkn1a | Cell cycle inhibitor | PMC11578798 |
| Cdkn1b | Cell cycle inhibitor | PMC11578798 |
| Il1b | SASP | 38654098 |
| Srgn | Senescence/aging marker | PMC10761730 |
| Clec4e | Senescence/aging marker | PMC9738698 |
| Malt1 | Senescence/aging marker | PMC7190641 |
| Cxcl2 | SASP | 38654098 |
| Plaur | Senescence/aging marker | PMC7583560 |
| Cd274 | Senescence/aging marker | 36323784 |
| Ptgs2 | Senescence/aging regulator | PMC8762039 |
| Pim1 | Senescence/aging regulator | PMC4331745 |
| Gadd45b | Cell cycle inhibitor | PMC11785585 |
| Mxd1 | Senescence/aging regulator | PMC102554 |
| Ets2 | Senescence/aging regulator | PMC7681055 |

Note: The genes marked in blue are present in the Banff Human Organ Transplant (B‐HOT) gene panel.

## **Table S5 The antibody information used in the research**

| Antibody | Type | Number | Firm |
| --- | --- | --- | --- |
| p21 | Recombinant antibody | GB15153-100 | Servicebio |
| CD68 | Recombinant antibody | GB113109-100 | Servicebio |
| NFKB | Recombinant antibody | GB11997-100 | Servicebio |
| uPAR | polyclonal antibody | GB112135-100 | Servicebio |
| Lrp2 | polyclonal antibody | GB112109-100 | Servicebio |
| Cd45 | Recombinant antibody | GB113886-100 | Servicebio |
