# Supplementary material for: Identification and characterization of senescent macrophages in renal allograft rejection: a cross-species MultiOmics study
Source: Front Immunol. 2025 Oct 9;16:1623124. doi: 10.3389/fimmu.2025.1623124 (PMC12545005; doi:10.3389/fimmu.2025.1623124)
Supplement: Supplementary file 2 [file Supplementaryfile2.pdf]

SUPPLEMENTARY 2

Figure legend

Figure S1 Boxplots showing the Expression of CS markers and core SASP components between non-rejection and rejection samples across multiple independent cohorts of the PROMAD dataset.

Figure S2 Boxplots comparing CSScores (A), CCAScore (B), and SenMayoScore (C) across Banff lesion score: interstitial inflammation (i), intimal arteritis (v), tubulitis (t), interstitial fibrosis (ci), tubular atrophy (ct), Glomerulitis (g), Peritubular Capillaritis (ptc), C4d (c4d), GBM Double Contours (cg), and Vascular Fibrous Intimal Thickening (cv) (Kruskal-Walli's test, all $p<0.001$). The results showing positive correlation between senescence signature scores with Banff pathology lesion grades.

Figure S3 HE staining showing significant interstitial and perivascular infiltration of hyperchromatic cells in allo-group kidneys versus syn controls, exhibiting concurrent glomerulonephritis, tubulointerstitial nephritis, and arteritis.

Figure S4 A Immunohistochemical staining of p21 in syn-graft (Syn) and allograft (Allo) renal tissues (scale bar: 100 μm). Inset: Regional comparison showing stronger $p21^+$ signal in tubulointerstitial compartments versus glomeruli. **B** β-galactosidase staining (blue) highlighting preferential senescence marker accumulation in tubulointerstitial regions of allografts. **C** Immunofluorescence detection of p21 (red) with DAPI nuclear counterstain (blue),

confirming predominant tubulointerstitial localization in allografts.

Figure S5 Immunofluorescence co-staining for p21 (red) with tubular marker Lrp2 (cyan) in allografts compared to syn-grafts (Scale bar 50μm). Quantitative analysis revealed no significant difference in $p21^{+}Lrp2^{+}$ tubular cell counts between allograft and syn-graft groups ($P > 0.05$).

Figure S6 Scatter plots showing the correlation between SenMayo GSVA score and infiltration of CD4+ T cells, CD8+ T cells, NK cells, B cells, dendritic cells (DCs), neutrophils, and mast cells (Pearson's test). The blue regression curve represents the fitted linear relationship between variables, and the blue shaded areas on both sides of the curve indicate the 95% confidence interval.

Figure S7 Scatter plots showing the correlation between M0 and M2 macrophages infiltration and three senescence GSVA scores (Pearson's test). The blue regression curve represents the fitted linear relationship between variables, and the blue shaded areas on both sides of the curve indicate the 95% confidence interval.

Figure S8 A Umap illustrating 20 distinct cell clusters from 2 rejection and 2 naïve cases of mouse scRNA-seq dataset1. PT, proximal tubular cell; Mon-Mac, monocyte derived macrophage (infiltrating macrophage); Res-Mac, resident macrophage; DCT, distal coiled tubule; LOH, loop of henle cells; Tfh, helper T cells; Endo, endothelium cells; CD-PC,

collecting duct principal cells; Prolif, proliferating cells; Mes, mesangial cells; CD-IC, collecting duct intercalated cell; pDC, plasmacytoid dendritic cell; Neu, neutrophile. **B** Proportion of each cell clusters in samples. **C** Dotplot representing expression and percentage of marker genes of each cell clusters. **D** Heatmap illustrating the distribution preferences of cell clusters across samples (indicated by OR values). **E** UMAP plots of cell clusters colored by SenMayo_signature and Senescence_signature, with violin plots comparing the signature scores across samples (two sided Wilcox-test, **** $p<0.0001$). F Dotplot showing enrichment scores of SenMayo_signature and Senescence_signature of alloimmune cells across samples, with macrophages highlighted (red frame).

Figure S9 Mouse single-cell dataset 2 A UMAP plots of infiltrating macrophages colored by SenMayo_signature, Senescence_signature and senescent cells. **B** Violin plots showing ssgsva scores of senescence gensets (GOBP: STRESS INDUCED PREMATURE SENESCENCE, GOBP: CELL CYCLE ARREST, REACTOME: SENESCENCE-ASSOCIATED SECRETORYPHENOTYPE) of senescent and not senescent IMs (two sided Wilcox-test, **$p<0.01$, **** $p<0.0001$). **C** Dot plot indicating differentially expressed genes (DEGs) between senescent and not senescent IMs. Genes with a $p < 0.01$ and logFC > 0.5 are shown highlighted as upregulated (red) in senescent IMs. **D** Bar plots displaying enrichment analysis and transcription factor prediction (**E**) of upregulated DEGs (up-DEGs) in senescent IMs, using Metascape and Trust database. **Human single-cell dataset F** UMAP plots of infiltrating macrophages colored by SenMayo_signature, Senescence_signature and senescent cells. **G** Bar plots showing proportion of senescent and not senescent IMs between rejection samples. **H**

Violin plots showing ss-gsva scores of senescence gensets (GOBP: STRESS INDUCED PREMATURE SENESCENCE, GOBP: CELL CYCLE ARREST, REACTOME: SENESCENCE-ASSOCIATED SECRETORYPHENOTYPE) of senescent and not senescent IMs (two sided Wilcox-test, **$p<0.01$, **** $p<0.0001$). **I** Dot plot indicating differentially expressed genes (DEGs) between senescent and not senescent IMs. Genes with a $p < 0.01$ and logFC > 0.5 are shown highlighted as upregulated (red) in senescent IMs. **J** Bar plots displaying enrichment analysis and transcription factor prediction (**K**) of upregulated DEGs (up-DEGs) in senescent IMs, using Metascape and Trust database.

Figure S10 Boxplots showing the expression levels (in FPKM) of SnIM signature genes (Cdkn1a, Cdkn1b, Il1b, etc.) in mouse bone marrow-derived macrophages (BMDMs) under different polarization conditions: unpolarized macrophages (M0, red), and macrophages polarized (LPS/IFN-γ 2h) to the M1 phenotype post 12 hours (M1_12h, green) and 24 hours (M1_24h, blue) (two sided Wilcox-test, **$p<0.01$, **** $p<0.0001$). The sample size for each group was $n = 3$.

Figure S1

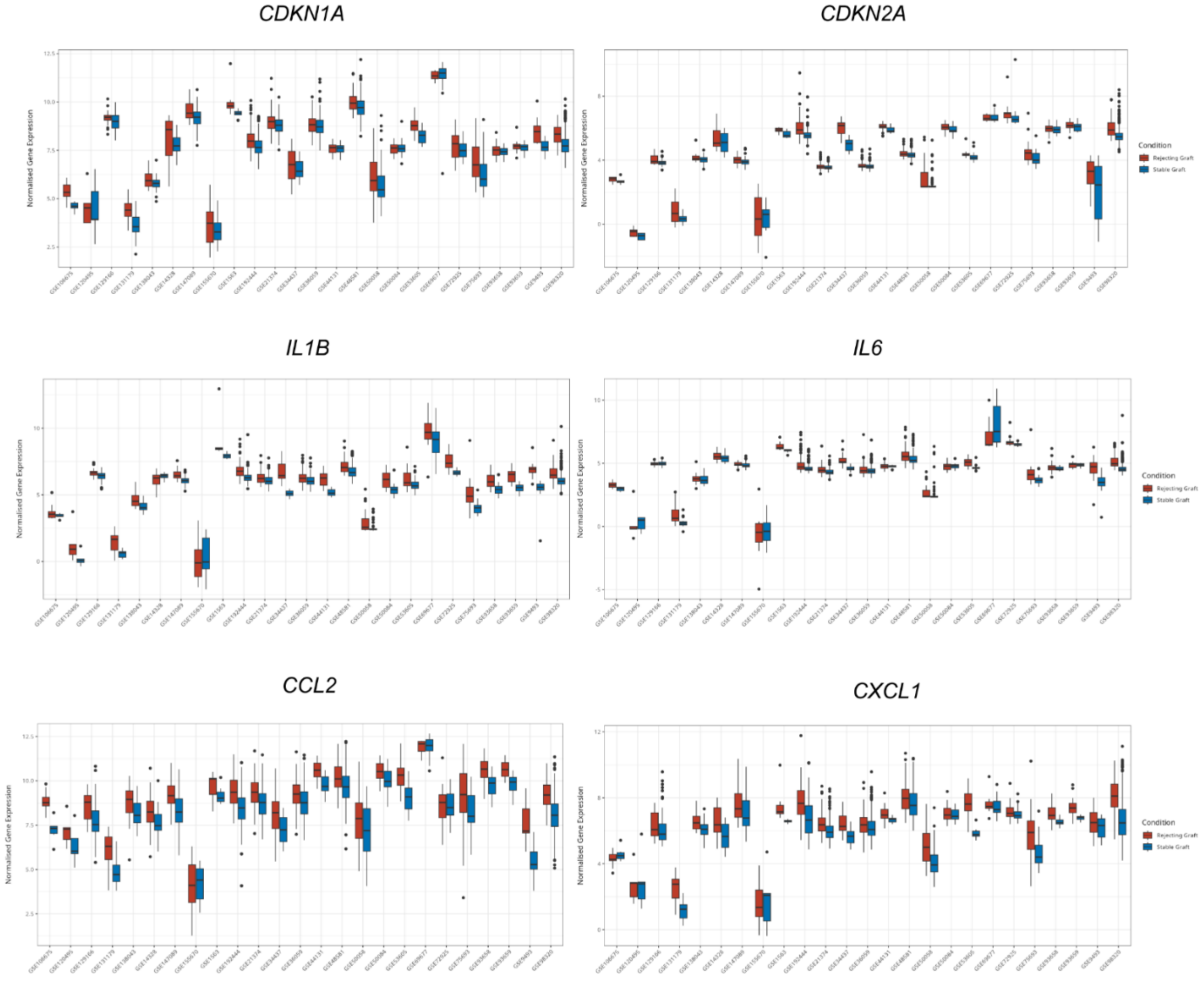
CDKN1A
CDKN2A
IL1B
IL6
CCL2
CXCL1
Normalised Gene Expression
Condition
Rejecting Graft
Stable Graft

Figure S2

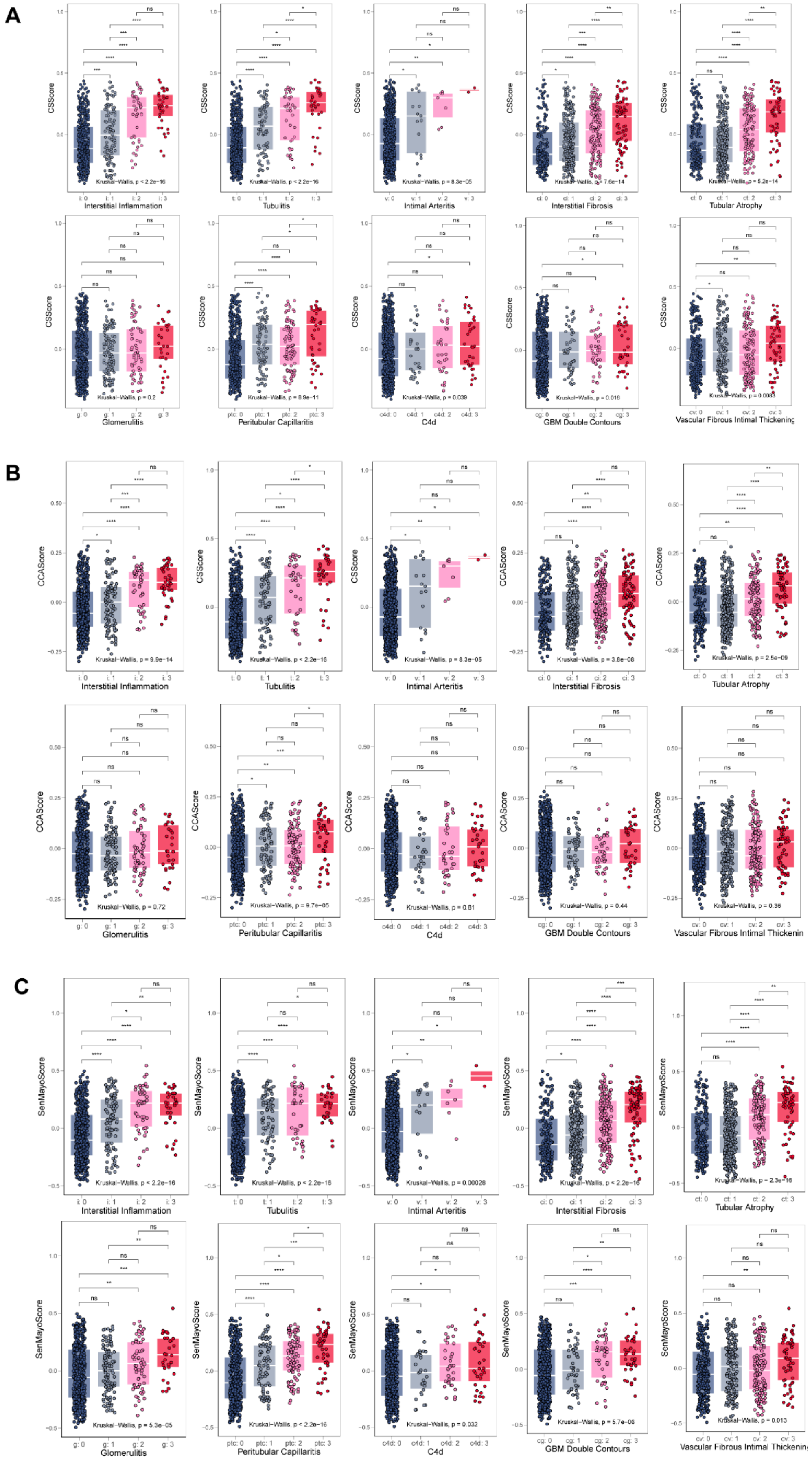
A
CSScore
Interstitial Inflammation
Tubulitis
Intimal Arteritis
Interstitial Fibrosis
Tubular Atrophy
Glomerulitis
Peritubular Capillaritis
C4d
GBM Double Contours
Vascular Fibrous Intimal Thickening
B
CCAScore
CSScore
Interstitial Inflammation
Tubulitis
Intimal Arteritis
Interstitial Fibrosis
Tubular Atrophy
Glomerulitis
Peritubular Capillaritis
C4d
GBM Double Contours
Vascular Fibrous Intimal Thickenin
C
SenMayoScore
Interstitial Inflammation
Tubulitis
Intimal Arteritis
Interstitial Fibrosis
Tubular Atrophy
Glomerulitis
Peritubular Capillaritis
C4d
GBM Double Contours
Vascular Fibrous Intimal Thickening

Figure S3

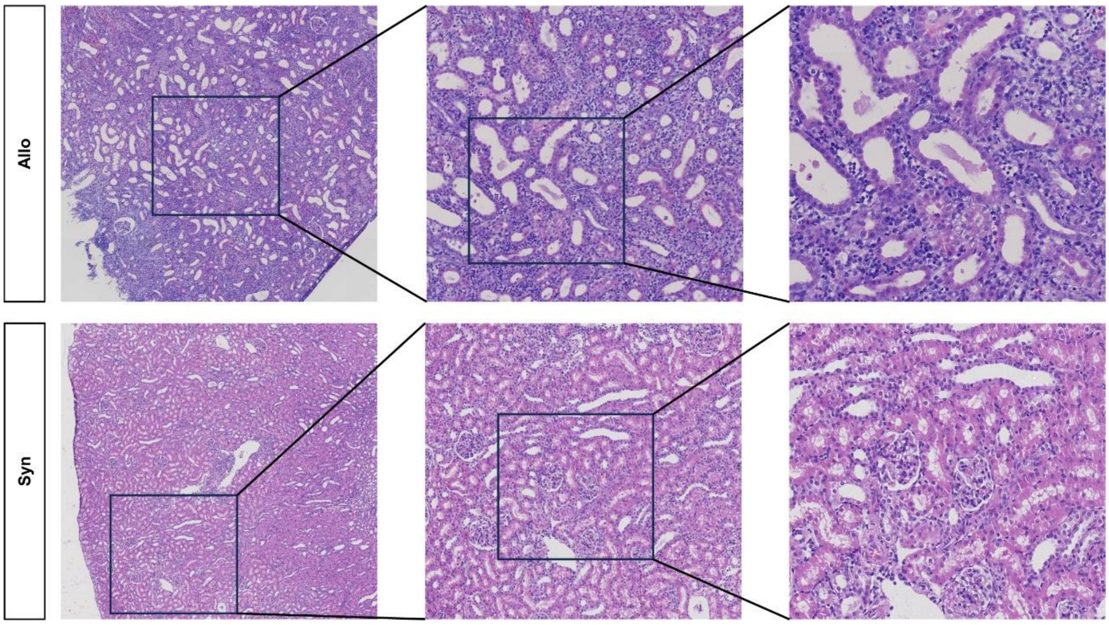

Figure S4

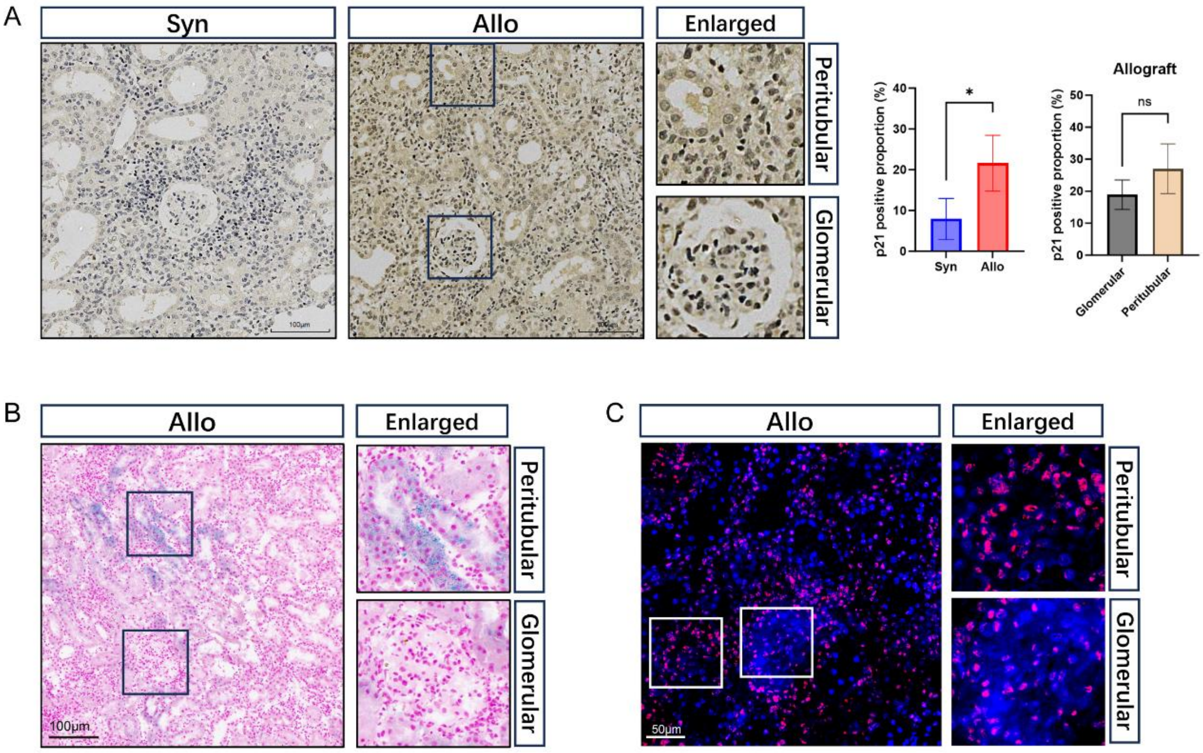

Figure S5

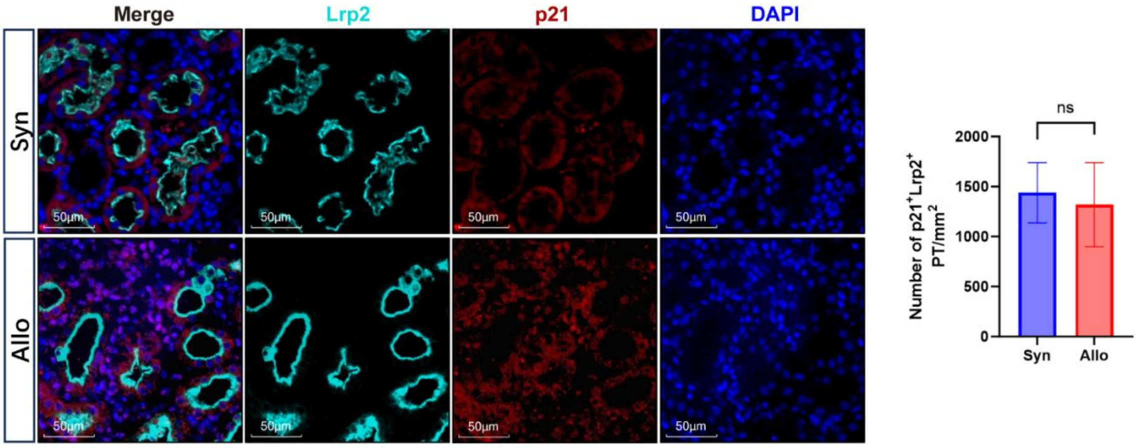

Figure S6

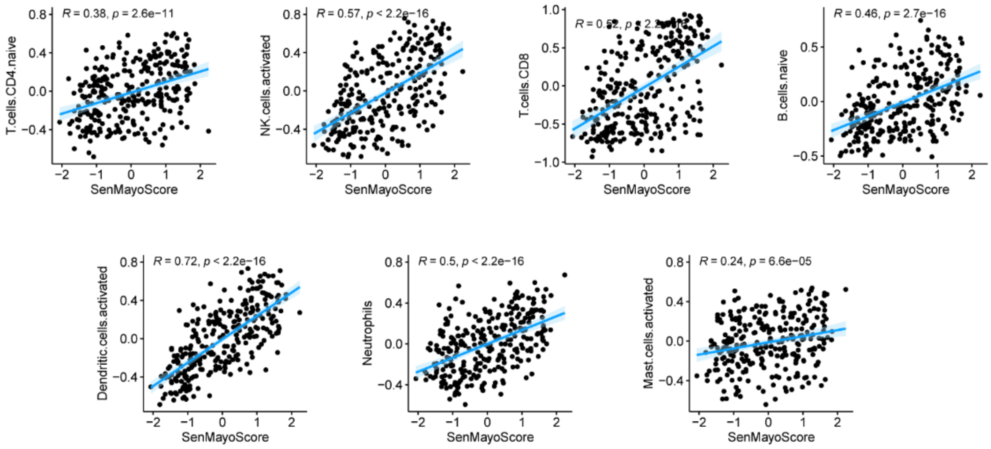

Figure S7

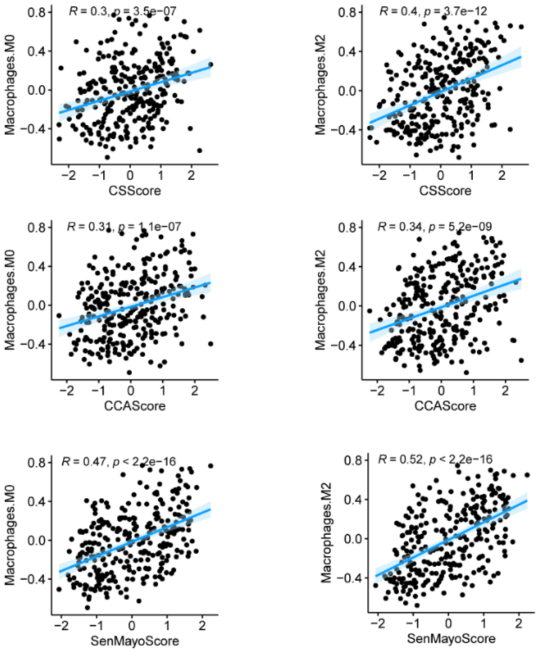

Figure S8

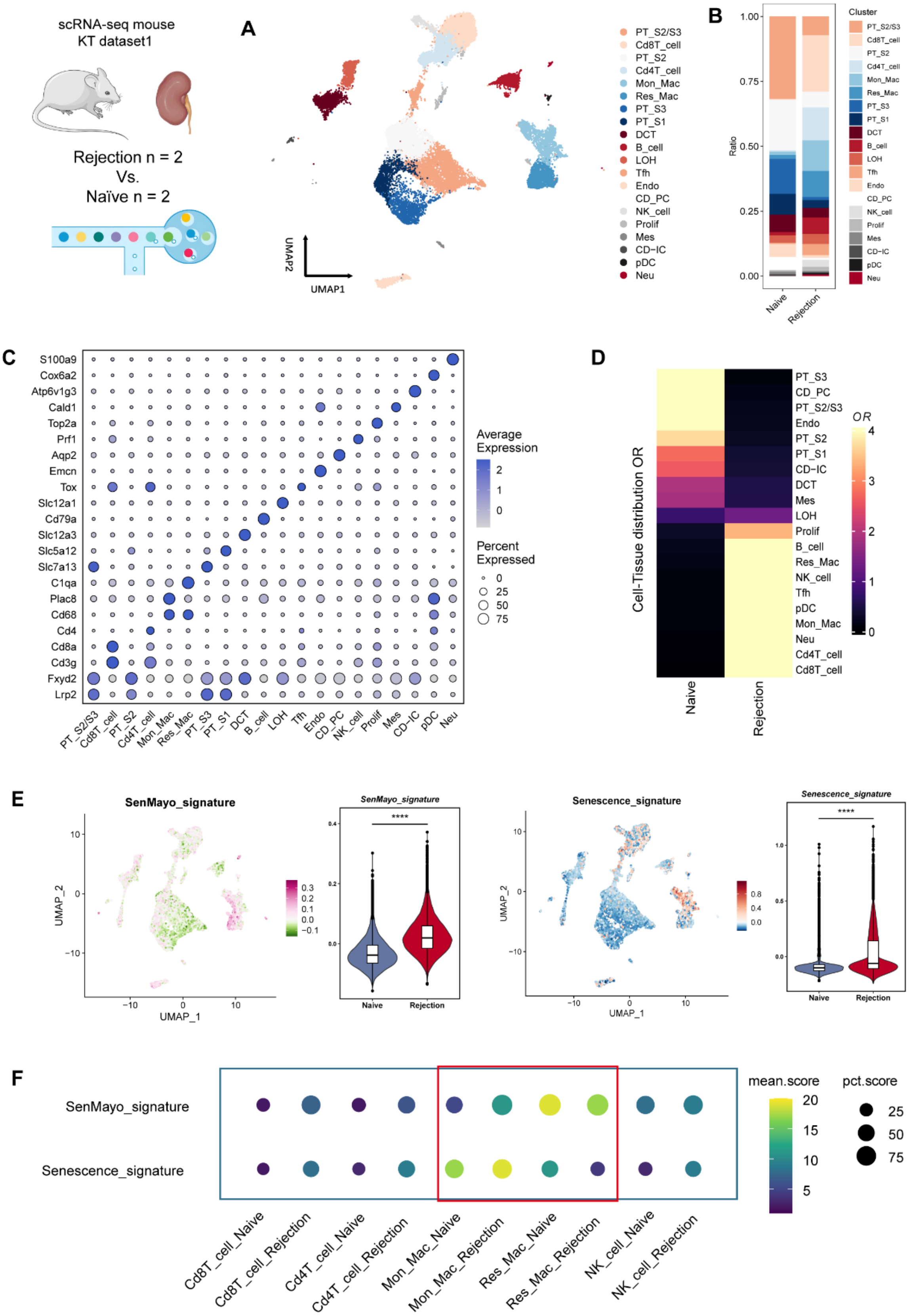
A
scRNA-seq mouse
KT dataset1
Rejection n = 2
Vs.
Naïve n = 2
UMAP1
UMAP2
PT_S2/S3
Cd8T_cell
PT_S2
Cd4T_cell
Mon_Mac
Res_Mac
PT_S3
PT_S1
DCT
B_cell
LOH
Tfh
Endo
CD_PC
NK_cell
Prolif
Mes
CD−IC
pDC
Neu
B
Ratio
Cluster
Naive
Rejection
C
S100a9
Cox6a2
Atp6v1g3
Cald1
Top2a
Prf1
Aqp2
Emcn
Tox
Slc12a1
Cd79a
Slc12a3
Slc5a12
Slc7a13
C1qa
Plac8
Cd68
Cd4
Cd8a
Cd3g
Fxyd2
Lrp2
Average Expression
Percent Expressed
D
Cell-Tissue distribution OR
OR
E
SenMayo_signature
Senescence_signature
UMAP_1
UMAP_2
Naive
Rejection
****
F
mean.score
pct.score
Cd8T_cell_Naive
Cd8T_cell_Rejection
Cd4T_cell_Naive
Cd4T_cell_Rejection
Mon_Mac_Naive
Mon_Mac_Rejection
Res_Mac_Naive
Res_Mac_Rejection
NK_cell_Naive
NK_cell_Rejection

FigureS9

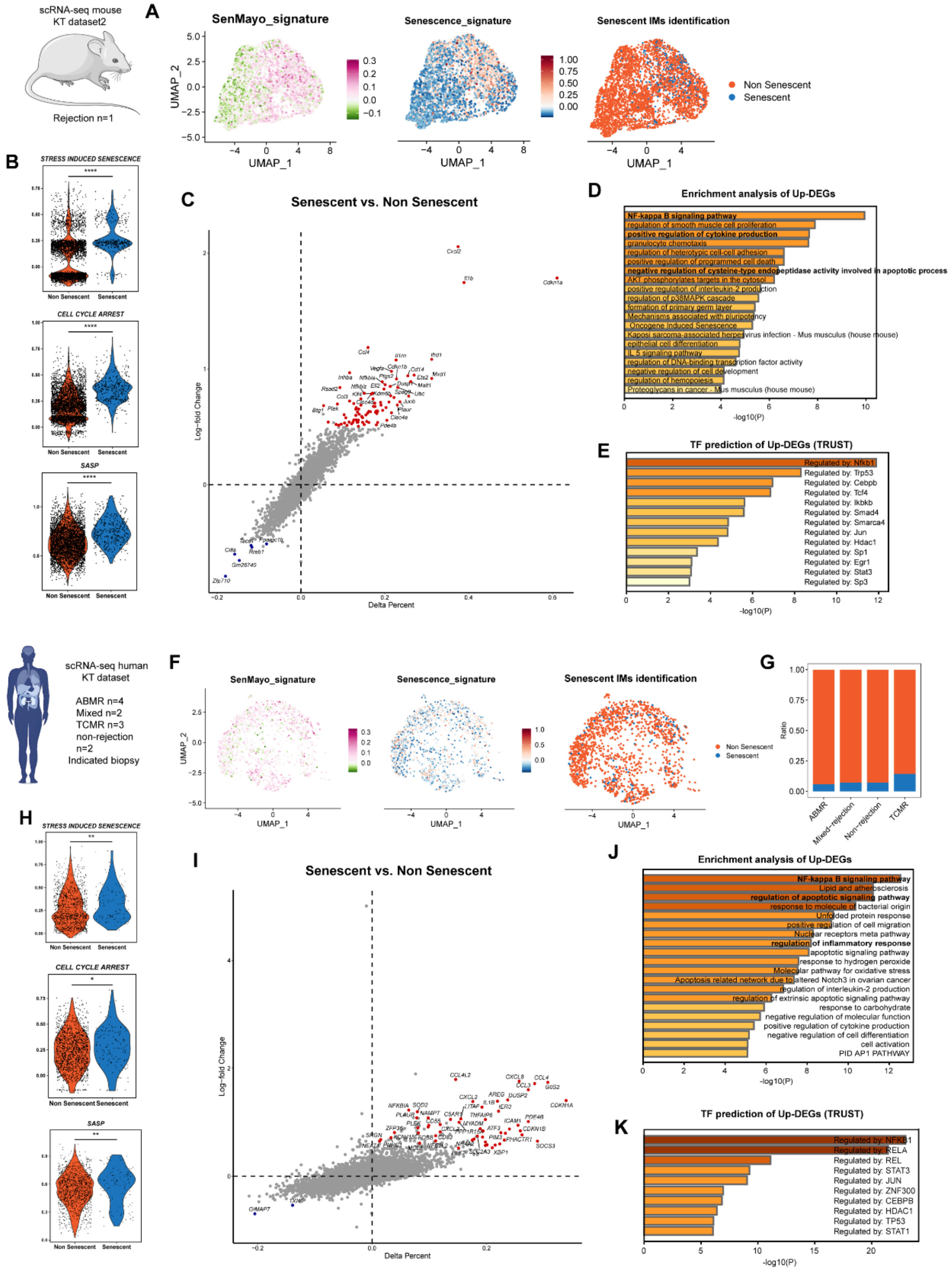
scRNA-seq mouse KT dataset2
Rejection n=1
A
SenMayo_signature
Senescence_signature
Senescent IMs identification
UMAP_1
UMAP_2
Non Senescent
Senescent
B
STRESS INDUCED SENESCENCE
CELL CYCLE ARREST
SASP
C
Senescent vs. Non Senescent
Log-fold Change
Delta Percent
D
Enrichment analysis of Up-DEGs
NF-kappa B signaling pathway
regulation of smooth muscle cell proliferation
positive regulation of cytokine production
granulocyte chemotaxis
regulation of heterotypic cell-cell adhesion
positive regulation of programmed cell death
negative regulation of cysteine-type endopeptidase activity involved in apoptotic process
AKT phosphorylates targets in the cytosol
positive regulation of interleukin-2 production
regulation of p38MAPK cascade
formation of primary germ layer
Mechanisms associated with pluripotency
Oncogene Induced Senescence
Kaposi sarcoma-associated herpesvirus infection - Mus musculus (house mouse)
epithelial cell differentiation
IL-5 signaling pathway
regulation of DNA-binding transcription factor activity
negative regulation of cell development
regulation of hemopoiesis
Proteoglycans in cancer - Mus musculus (house mouse)
-log10(P)
E
TF prediction of Up-DEGs (TRUST)
Regulated by: Nfkb1
Regulated by: Trp53
Regulated by: Cebpb
Regulated by: Tcf4
Regulated by: Ikbkb
Regulated by: Smad4
Regulated by: Smarca4
Regulated by: Jun
Regulated by: Hdac1
Regulated by: Sp1
Regulated by: Egr1
Regulated by: Stat3
Regulated by: Sp3
scRNA-seq human KT dataset
ABMR n=4
Mixed n=2
TCMR n=3
non-rejection n=2
Indicated biopsy
F
G
Ratio
ABMR
Mixed-rejection
Non-rejection
TCMR
H
I
J
Enrichment analysis of Up-DEGs
NF-kappa B signaling pathway
Lipid and atherosclerosis
regulation of apoptotic signaling pathway
response to molecule of bacterial origin
Unfolded protein response
positive regulation of cell migration
Nuclear receptors meta pathway
regulation of inflammatory response
apoptotic signaling pathway
response to hydrogen peroxide
Molecular pathway for oxidative stress
Apoptosis related network due to altered Notch3 in ovarian cancer
regulation of interleukin-2 production
regulation of extrinsic apoptotic signaling pathway
response to carbohydrate
negative regulation of molecular function
positive regulation of cytokine production
negative regulation of cell differentiation
cell activation
PID AP1 PATHWAY
K
TF prediction of Up-DEGs (TRUST)
Regulated by: NFKB1
Regulated by: RELA
Regulated by: REL
Regulated by: STAT3
Regulated by: JUN
Regulated by: ZNF300
Regulated by: CEBPB
Regulated by: HDAC1
Regulated by: TP53
Regulated by: STAT1

Figure S10

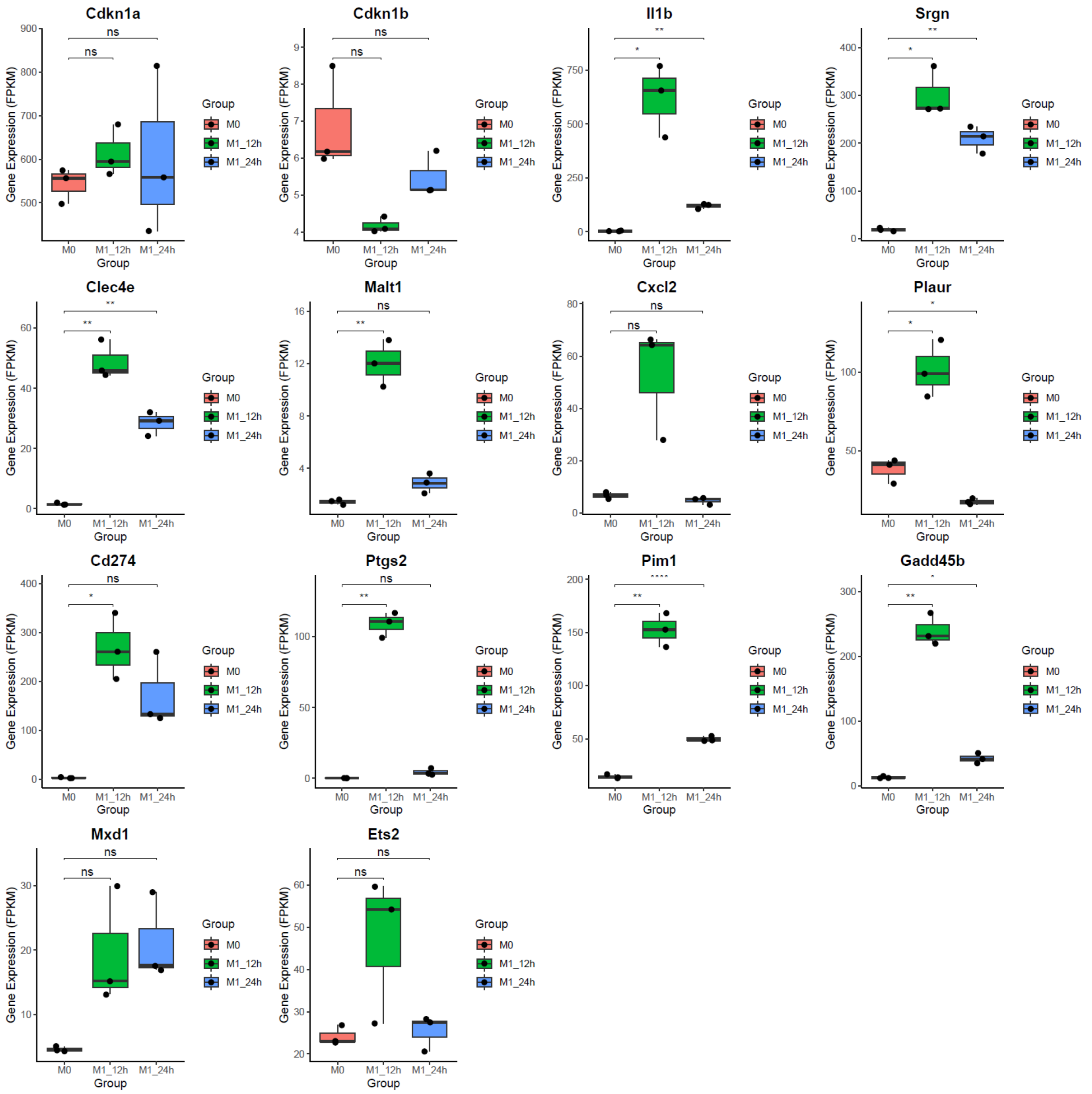
Cdkn1a
Cdkn1b
Il1b
Srgn
Clec4e
Malt1
Cxcl2
Plaur
Cd274
Ptgs2
Pim1
Gadd45b
Mxd1
Ets2
Gene Expression (FPKM)
Group
M0
M1_12h
M1_24h
ns
