# Supplementary material for: Identification and characterization of senescent macrophages in renal allograft rejection: a cross-species MultiOmics study
Source: Front Immunol. 2025 Oct 9;16:1623124. doi: 10.3389/fimmu.2025.1623124 (PMC12545005; doi:10.3389/fimmu.2025.1623124)
Supplement: Supplementary file 3 [file Table1.docx]

##Clearing the environment

rm(list=ls())

setwd("~")

###Loading the required packages

library(Seurat)

library(tidyverse)

library(dplyr)

library(patchwork)

###Loading single-cell data

load('~/Seurat.object.Rdata')

###################Senescence_signature###################################

senescence_marker= c("Cdkn2a","Cdkn1a", "Serpine1" ,"Cdkn1b", "Cdkn2d", "Cdkn2b")

VlnPlot(Seurat.object, senescence_marker)

Seurat.object=AddModuleScore(Seurat.object, features = list(senescence_marker))

Seurat.object$Cluster1-> Seurat.object$Senescence_signature

library(RColorBrewer)

library(scales)

FeaturePlot(Seurat.object, features = "Senescence_signature", pt.size = 0.6)+

scale_color_gradientn(colours = rev(brewer.pal(n = 11, name = "RdBu")),

values=rescale(c(min(Seurat.object$Senescence_signature),0,max(Seurat.object$Senescence_signature))),guide="colorbar",limits=c(min(Seurat.object$Senescence_signature),max(Seurat.object$Senescence_signature)))

####################SenMayo signature############################################

sengene <-read.table("SenMayo signature.txt", header=F, sep="\t", check.names=F)

Genset <- sengene$V1

SenMayo= c(Genset)

Seurat.object=AddModuleScore(Seurat.object, features = list(SenMayo))

Seurat.object$Cluster1-> Seurat.object$SenMayo_signature

FeaturePlot(Seurat.object, features = "SenMayo_signature", pt.size = 0.6)+

scale_color_gradientn(colours = rev(brewer.pal(n = 11, name = "PiYG")),

values = rescale(c(min(Seurat.object$SenMayo_signature),0,max(Seurat.object$SenMayo_signature))),guide="colorbar",limits=c(min(Seurat.object$SenMayo_signature),max(Seurat.object$SenMayo_signature)))

####################Identification of senescent cells############################

quantile<-cbind(as.character(cut(as.numeric(as.character(Seurat.object$Senescence_signature)),as.numeric(quantile(Seurat.object$Senescence_signature)))),as.character(cut(as.numeric(as.character(Seurat.object$SenMayo_signature)),as.numeric(quantile(Seurat.object$SenMayo_signature)))))

colnames(quantile)<-c("Senescence_scoreSENQUANTILE", "Senescence_scoreSMQUANTILE")

rownames(quantile)<-colnames(Seurat.object)

quantile<-as.data.frame(quantile)

quantile$Senescence_signature<-Seurat.object$Senescence_signature

quantile$SenMayo_signature<-Seurat.object$SenMayo_signature

table(quantile$Senescence_scoreSENQUANTILE)

quantile[which(is.na(quantile$Senescence_scoreSENQUANTILE)),]

quantile$Senescence_groupSENQUANTILE=NA

quantile[which((quantile$Senescence_scoreSENQUANTILE)%in%"(min, ]"),]$Senescence_groupSENQUANTILE="quantile1"

quantile[which((quantile$Senescence_scoreSENQUANTILE)%in%"( , ]"),]$Senescence_groupSENQUANTILE="quantile2"

quantile[which((quantile$Senescence_scoreSENQUANTILE)%in%"( , ]"),]$Senescence_groupSENQUANTILE="quantile3"

quantile[which((quantile$Senescence_scoreSENQUANTILE)%in%"( ,max]"),]$Senescence_groupSENQUANTILE="quantile4"

table(quantile$Senescence_scoreSMQUANTILE)

quantile[which(is.na(quantile$Senescence_scoreSMQUANTILE)),]

quantile$Senescence_groupSMQUANTILE=NA

quantile[which((quantile$Senescence_scoreSMQUANTILE)%in%"(min, ]"),]$Senescence_groupSMQUANTILE="quantile1"

quantile[which((quantile$Senescence_scoreSMQUANTILE)%in%"( , ]"),]$Senescence_groupSMQUANTILE="quantile2"

quantile[which((quantile$Senescence_scoreSMQUANTILE)%in%"( , ]"),]$Senescence_groupSMQUANTILE="quantile3"

quantile[which((quantile$Senescence_scoreSMQUANTILE)%in%"( ,max]"),]$Senescence_groupSMQUANTILE="quantile4"

quantile$ALL4QUANTILE<-"Non Senescent"

quantile[which((quantile$Senescence_groupSENQUANTILE %in% "quantile4") & (quantile$Senescence_groupSMQUANTILE%in%"quantile4")),]$ALL4QUANTILE<-"Senescent"

Seurat.object$SENEQUANTILE<-quantile[colnames(Seurat.object),]$ALL4QUANTILE

DimPlot(Seurat.object, group.by = "SENEQUANTILE",cols= c("Non Senescent"="#F15A2B" , "Senescent"="#1D75BC"))+ ggtitle("SENESCENT CELLS IDENTIFICATION")
